# Bacterial Membrane Mimetics: From Biosensing to Disease Prevention and Treatment

**DOI:** 10.3390/bios13020189

**Published:** 2023-01-26

**Authors:** Sagar S. Arya, Nada K. Morsy, Deema K. Islayem, Sarah A. Alkhatib, Charalampos Pitsalidis, Anna-Maria Pappa

**Affiliations:** 1 Department of Biomedical Engineering, Khalifa University of Science and Technology, Abu Dhabi P.O. Box 127788, United Arab Emirates; 2 Department of Physics Khalifa University of Science and Technology, Abu Dhabi P.O. Box 127788, United Arab Emirates; 3 Healthcare Engineering Innovation Center (HEIC), Khalifa University of Science and Technology, Abu Dhabi P.O. Box 127788, United Arab Emirates; 4 Department of Chemical Engineering and Biotechnology, Cambridge University, Philippa Fawcett Drive, Cambridge CB30AS, UK

**Keywords:** bacterial membranes, biosensing, nanoparticles, antimicrobial resistance, cancer, immunotherapy

## Abstract

Plasma membrane mimetics can potentially play a vital role in drug discovery and immunotherapy owing to the versatility to assemble facilely cellular membranes on surfaces and/or nanoparticles, allowing for direct assessment of drug/membrane interactions. Recently, bacterial membranes (BMs) have found widespread applications in biomedical research as antibiotic resistance is on the rise, and bacteria-associated infections have become one of the major causes of death worldwide. Over the last decade, BM research has greatly benefited from parallel advancements in nanotechnology and bioelectronics, resulting in multifaceted systems for a variety of sensing and drug discovery applications. As such, BMs coated on electroactive surfaces are a particularly promising label-free platform to investigate interfacial phenomena, as well as interactions with drugs at the first point of contact: the bacterial membrane. Another common approach suggests the use of lipid-coated nanoparticles as a drug carrier system for therapies for infectious diseases and cancer. Herein, we discuss emerging platforms that make use of BMs for biosensing, bioimaging, drug delivery/discovery, and immunotherapy, focusing on bacterial infections and cancer. Further, we detail the synthesis and characteristics of BMs, followed by various models for utilizing them in biomedical applications. The key research areas required to augment the characteristics of bacterial membranes to facilitate wider applicability are also touched upon. Overall, this review provides an interdisciplinary approach to exploit the potential of BMs and current emerging technologies to generate novel solutions to unmet clinical needs.

## 1. Introduction

Bacteria are single-celled organisms found in their millions in every (micro) environment, both inside and outside of other organisms. Their membrane, mainly comprised of proteins, sterols, and phospholipids, has been increasingly explored as a model for investigating interfacial phenomena at the first point of contact: the plasma membrane. Drawing inspiration from nature, bacterial membranes (BMs) with various complexity levels have been widely developed not only to test drug interactions but also for novel multifaceted therapies, given the multiple roles of bacteria in our body.

The unsupervised excessive antibiotic use has resulted in antibiotic resistance, which is currently outpacing the launch of new antimicrobials, leading to a global health crisis [1,2]. One strategy to accelerate the drug discovery pipeline is the development of accurate and fast assays to systematically test existing and newly developed drugs. BMs can be powerful test beds as they constitute an important barrier to protect against the external environment and mediate selective permeability of biomolecules/metabolites into and outside bacteria. As such, BMs are rapidly explored in conjunction with other technologies, including bioelectronics, nanomaterials, and microfluidics, resulting in high throughput label-free assays to test the efficacy of antibiotics, as well as to provide fundamental understanding of the factors that govern antibiotic/BMs interactions.

BMs have been also used to overcome some of the limitations of conventional cancer therapy, as bacteria can act as potent antitumor agents or be genetically engineered to release specific anti-cancer compounds and modify their metabolic pathways. Given their targeting capabilities, BM coated nanoparticles (BM-NPs) have been explored for immunotherapy or cancer treatment and have also served as bioinspired platforms for immunomodulation against existing bacterial infections or to prime the immune system against pathogenic bacteria [3,4,5,6].

Considering the growth in literature on animal cell membrane mimetics and limited information on bacterial membrane mimetics, this review summarizes the characteristics, synthesis, and isolation of BMs. It contextualizes the advances in the work on BMs and their future in the fields of biosensing, cancer therapy, drug discovery, and immunomodulation. Lastly, strategies to augment the characteristics of BMs and BM-NPs for broadening their application are highlighted.

## 2. Bacterial Membrane Characteristics

**Figure 1:**
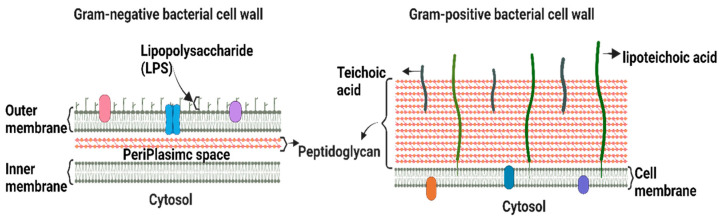
Schematic depiction of the key structural differences between Gram-positive and Gram-negative bacteria.

Bacteria are classified as Gram-positive and Gram-negative, differing in their structure and cell wall lipid composition (Figure 1). The main difference between the two categories is the lack of an outer membrane in the Gram-positive cell envelope [7].

Gram-positive bacteria are characterized by a thick cell wall surrounding the cytoplasmic membrane consisting of multiple peptidoglycan layers with a mesh-like frame [7]. This structure provides protection from the environment and maintains cell integrity and shape, allowing bacteria to withstand the osmotic and mechanical pressures exerted on the plasma membrane. Gram-positive bacteria are surrounded by a notably thicker murein wall (40–80 nm) compared to Gram-negative bacteria (7–8 nm) [8]. The membranes of Gram-positive bacteria are composed of phospholipids, lipid-anchoring components (lipoteichoic acid), lipoproteins, and transmembrane proteins [9].

The Gram-negative cell envelope consists of an inner membrane, a periplasm, and an outer membrane. The inner membrane is an asymmetric phospholipid bilayer that wraps around the cytosol [10,11]. The periplasm contains a thin layer of peptidoglycan that supports the shape and rigidity of the bacterium. The outer membrane is an asymmetric lipid bilayer that surrounds the periplasmic space [10]. The proximal leaflet is composed of phospholipids, whereas the distal leaflet is mostly composed of lipopolysaccharides (LPS) [10], which act as a protective barrier [12]. LPS is a glycolipid whose structure differs significantly between Gram-negative bacterial species owing to survival adaptations in response to changes in environmental stimuli, such as temperature, pH, certain ion concentrations, toxins, antibiotics, and osmotic pressure [13,14]. The outer membrane and LPS leaflets are absent in most Gram-positive bacteria [9]. Therefore, alterations in the outer membrane characteristics, such as hydrophobic properties, LPS, or mutations in porins and other factors, can create resistance, making Gram-negative bacteria more resistant to antibiotics than Gram-positive ones [15]. Hence, the majority of the WHO-listed pathogens are Gram-negative bacteria, which are the cause of significant morbidity and mortality worldwide.

Typical bacterial plasma membrane phospholipids include phosphatidylglycerol (PG), cardiolipin (CL), and phosphatidylethanolamine (PE) [10,11]. The composition varies greatly in different and within the same Gram-type bacterial species, where a high degree of structural, chemical, and functional variation exists in lipid membranes [16,17,18]. Therefore, many biomimetic bacterial membrane models have been developed over the last century to study their properties, structure, and physicochemical processes [19]. The three most common models are lipid vesicles (liposomes), lipid bilayers (SLBs), and lipid monolayers [20,21,22,23,24,25]. These systems mimic the lipid assembly of bacterial cell membranes to some extent, and each has advantages and limitations. Below, the main systems are addressed, along with examples of their application as bacterial biomimetic membranes.

## 3. Bacterial Membrane Vesicles (BMVs) to Mimic Bacterial Membranes

### 3.1. Natural BMVs

BMVs (as described and summarized in Table 1) play key roles in immune regulation in potential hosts [26], support bacterial biofilm formation [27] as well as participate in intracellular communication (i.e., horizontal gene transfer between different bacterial species) [28,29].

There are two main types of BMVs that are secreted not only in stressful but also under standard growth conditions. One of these is outer membrane vesicles (OMVs), which are secreted by the outer membrane of the complex cell envelope [30] that surrounds Gram-negative bacteria. The second type is Cytoplasmic Membrane Vesicles (CMVs), which are synthesized from Gram-positive bacteria and arise directly from their cytoplasmic membrane [31]. In addition to these typical BMVs, several other structures, such as explosive outer-membrane vesicles [32], tubular membrane structures [33], and outer-inner vesicles [34], are also secreted by bacteria.

**Table 1:** Structure, description, features, and applications of biomimetic membranes. [Langmuir–Blodgett (LB), Langmuir–Schaefer (LS)].

Model	Type	Features	Limitations	Applications	Ref.
**Vesicles** 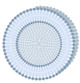 *Sphere-shaped lipid bilayers encasing an aqueous core*	*Natural* *Artificial*	-Stability, functionality, and mobility (proteins and ion channels) -Customizable (lipid/protein composition)	-Difficult to control lipid asymmetry -Complex & selective preparation techniques	-Investigate membrane phase behavior -Fusion -Molecular recognition -Cell adhesion -Drug delivery	[35,36,37,38,39]
**Monolayers** 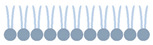 *Lipid monolayer representing half bilayer*	*LB*	-Stability -Allow molecular packing, thermodynamic analysis, and insertion of amphipathic compounds	-Lacks complexity of native membrane -Lacks lateral mobility	-Study lipid packing -2D surface phenomena -Adsorption, wetting, phase transitions -Drug interaction studies	[19,40,41,42,43]
**Supported lipid bilayers (SLBs)** 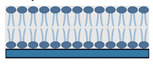 *SLBs represent two leaflets of a BM supported on a substrate*	*Vesicle fusion/* *(LB/LS)*	-Biomimetic -Controllable asymmetry	-Less stable in air compared to monolayers -Restricted customization, mobility of membrane components	-Molecular interactions, lateral topography, lipid mixing, dynamics/diffusion -Study phase behavior -Mimic models that associate peripheral proteins	[35]
**Free ‘floating’ SLBs** 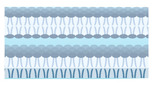 *SLB deposited on bilayer, LB, or hybrid bilayer separated by a medium*	*Vesicle fusion/* *(LB/LS)*	-Represent realistic fluid membrane -Customizable	-Stability depends on pH, ionic strength, and gel phase -Time and expertise dependent	-Develop molecular systems based on membrane fluidity -Protein integration -Design drug carriers/biosensors -Membrane-cell fusion -Molecular recognition	[35,44,45]
**Tethered SLBs** 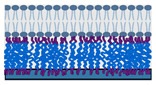 *Bilayer covalently linked to hydrophilic tethers/polymer cushions*	*Vesicle fusion/* *(LB/LS)*	-Customizable -Reduced lipid-substrate interaction, stable compared to Free “Floating” SLBs -Controllable mobility, structure, and electrical sealing properties	-High stability restricts lateral lipid mobility	-Vesicle fusion via vesicle-polymer electrostatic attraction -Physicochemical, structural, electrical, and ionic evaluation -Drug testing and sensing	[35,46,47,48,49,50]

In Gram-positive bacteria, cytoplasmic membrane vesicles are thought to arise from either dying cells or other conserved vesicle mechanisms [51]. However, owing to the thick peptidoglycan layer, the effects of membrane instability are relatively negligible, leading to the protrusion of the cytoplasmic membrane through the pores in the peptidoglycan layer, ultimately releasing CMVs.

On the other hand, in Gram-negative bacteria, OMVs are formed when part of the outer membrane detaches from the bacterial surface and encapsulates a part of the periplasmic space. These OMVs are composed of LPS, phospholipids, proteins, and nucleic acids [52]. The synthesis of OMVs in Gram-negative bacteria is thought to occur because of swelling in areas with impaired peptidoglycan connections, which are disrupted either by breaking or repositioning the links [53,54]. Further budding of the bulge also occurs due to the accumulation of periplasmic proteins in the bacterial cell wall, leading to the dissociation of the outer membrane.

BMV production occurs through the formation of various defects that are triggered by several genetic and environmental factors, such as disturbance of cell-envelope crosslinking (between the peptidoglycan and the outer membrane) [53,54], “bilayer coupling” effects that are obtained by molecules that yield membrane curvatures, and accumulation of misfolded proteins in the periplasmic space [55,56]. An example of this mechanism is shown in one of the most explored hypervesiculating bacteria, *E. coli* JC8031; the vesicles are produced due to the genetic knockout of the *tolRA* gene, causing membrane instability in the *E. coli* cell envelope [57].

Other environmental factors, including bacterial growth conditions, stress factors (including thermal stress and antibiotic stress), and media composition, can also trigger the release of large amounts of BMVs [37]. Another possible mechanism for vesicle blebbing is the degradation of the peptidoglycan layer, catalyzed by the action of the autolysin/endolysin enzyme. For example, in Gram-negative bacteria, endolysins are used to destabilize the membrane, leading to explosive cell lysis and vesicle formation [58].

The most common vesicle formation process usually consists of the following steps: The first step is to control the cultivation in the liquid medium, the composition of the medium, the timing of harvesting, and the application of different stress conditions that affect the vesicle formation rate and OMVs content [35]. Slow centrifugation is then applied to remove most of the intact bacteria, and the remainder is then removed by sterile filtration. The filtrate is then preconcentrated before centrifugation, either by precipitation or ultrafiltration [52]. Purification procedures, such as sucrose gradient centrifugation and size exclusion chromatography, are then used to remove proteins not associated with OMVs. At each step of the workflow, the OMVs yield can be quantified, and its stability and associated bioactivity can be monitored, which varies based on different isolation methods used [52].

Another purification (isolation) procedure depends on the bacterial membrane features, where positively charged antimicrobial substances such as ϵ-Poly-L-lysine are used to capture extracellular vesicles and precipitate them, depending on the amounts of negatively charged LPS and lipoteichoic acid that are found in extracellular vesicles produced from Gram-negative and Gram-positive bacteria, respectively [59]. Besides, immunoaffinity chromatography extraction methods are used for isolating OMVs based on the recognition of a specific ligand binding to its target molecule, where Alves et al. [60] suggested the insertion of a specific 6xHis-taq sequence that binds to the exterior facing a highly expressed protein domain (OmpA) usually found on BMVs to act as a biomarker for selective, direct, and rapid isolation by immobilized metal affinity chromatography. Huang et al. [61] provided a comprehensive understanding of OMV biogenesis, isolation, purification, and application in vaccine and immunoadjuvant development.

### 3.2. Artificial BMVs: Liposome Techniques

Liposomes is another term used to describe natural and artificial spherical-shaped vesicles that are comprised of one or more phospholipid bilayers that encase an aqueous core [35]. In this section, liposomes are used to refer to the artificially synthesized BMVs.

Liposomes are the preferred structures adopted by bilayers when in contact with water. This is because it adopts a lower, more energetically stable form in this configuration, where the polar head groups are in contact with the liquid interface while the hydrocarbon tails remain within the bilayer [35]. The size of synthesized liposomes usually ranges from nanometers to microns in diameter [62]. Their lamellar structures and properties vary depending on the manufacturing process, lipophilic composition, functionalization, and surface charge, allowing for a significant level of customization [35]. They are usually classified according to vesicle size and lamellar structure [63], where unilamellar vesicles are small (20–40 nm), medium (40–80 nm), large (100–1000 nm), or even larger (>1000 nm) [62]. Oligolamellar vesicles are composed of 2–10 bilayers, and multilamellar vesicles have multiple bilayers.

**Figure 2:**
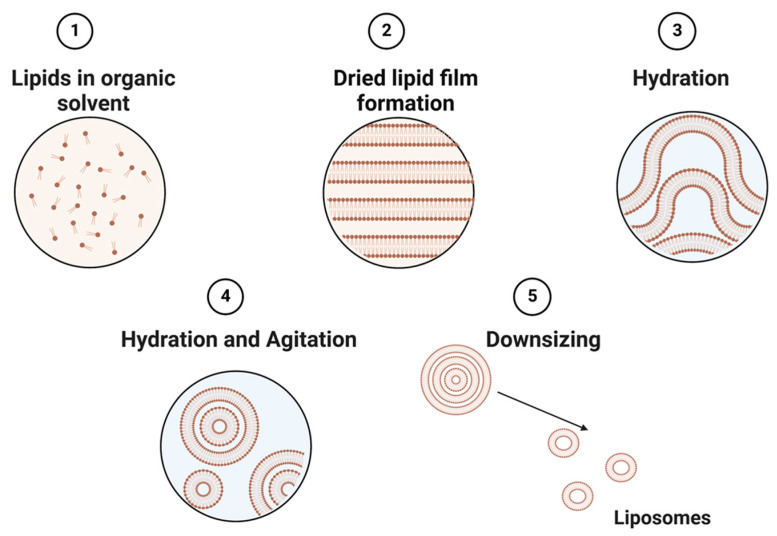
Schematic representation of the main stages of the thin film hydration method of liposome preparation.

Liposomes can be obtained by several processes, including mechanical techniques, organic solvents, or detergent removal [35]. The simplest and most common approach to constructing liposomes is to utilize different types of thin-film hydration techniques (Figure 2) [63]. This technique utilizes a physical dispersion method, which involves solvent evaporation followed by rehydration in the aqueous phase prior to downsizing and homogenization. The size of liposomes is influenced by their lipid charges and aqueous phase properties [64]. Alternatively, the solvent dispersion method could be applied, where lipids in the solvent are mixed with the aqueous phase containing the elements to be encapsulated [38]. Other methods, such as detergent solubilization, are also used, where micelle–vesicle transition detergents are used to solubilize lipids in micellar systems [38]. These lipids can then be released by dilution, membrane filtration, hollow fiber dialysis, gel chromatography, or adsorption onto a hydrophobic matrix (resins) [65].

In a study by Drost et al. [66], liposomes composed of dioleoyl phosphatidylglycerol (DOPC) and CL were generated by thin film hydration, resembling the cell membranes of Gram-positive *S. aureus* and *S. pneumoniae*. It was purified using size-exclusion chromatography. This bacterial mimetic liposome was created to provide a suitable model for drug release, liposome–cell interactions, and a better encapsulation efficiency for a highly hydrophobic antibiotic.

Regardless of whether they are artificially synthesized or directly triggered by cells, all types of BMVs have the same strengths and weaknesses stemming from their structure-function properties. For example, because vesicles/liposomes are composed of two lipid sheets, they mimic bacterial biological membranes better than Langmuir monolayers (discussed later) and provide an excellent model for studying membrane phase behavior and other membrane processes [35], as further mentioned in Table 1. However, these preparation techniques are complex and selective. In addition, when using vesicle model systems, it is more difficult to control lipid asymmetry. Therefore, the final composition of the produced vesicles should always be examined before direct use, as it may differ from the composition of the initial lipid mixture used to form vesicles or from the original cell membrane [62].

## 4. Lipid Layers to Mimic the Bacterial Membrane

### 4.1. Langmuir-Blodgett

**Figure 3:**
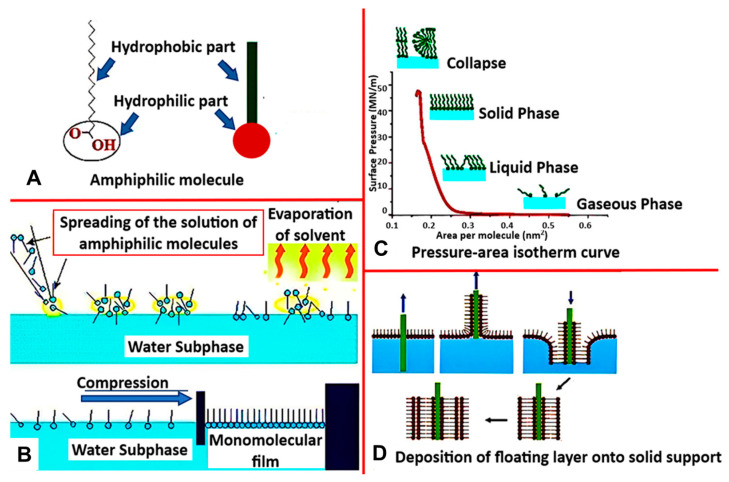
(**A**) Structure of amphiphilic molecule with hydrophobic and hydrophilic parts, (**B**) Langmuir Blodget process (**C**) Isotherm curve representing thermodynamic behavior of Langmuir-monolayer (**D**) Method of deposition of floating layer on to a solid support. [reproduced with permission from Hussain et al. [67], copyright 2018 Cell Press].

One of the most common biomimetic systems used to recreate natural membranes is the Langmuir monolayer (LM) [35]. This lipid monolayer is a simplified model formed via the Langmuir-Blodgett technique, which utilizes the amphiphilic properties of lipids to orient themselves in a liquid/air interface (Figure 3A). As the lipid molecules are deposited on the surface, dispersion forces immediately cause the solution to spread over with the lipid headgroups submerged into the subphase, and hydrophobic tails point in the direction of the gaseous phase. This results in forming a monomolecular layer interfacial film (Figure 3B), which gets compressed by movable barriers along the water’s surface. The surface pressure can then be constantly recorded as a function of the mean molecular area to provide more information about the lipid–lipid, lipid–water, lipid–protein, or lipid–drug interactions (Figure 3C). 

This technique is increasingly recognized in basic biodegradation research for understanding the mechanism of action of microorganisms. It has been widely used as a membrane model because of its uniformity, planar shape, and specific orientation, and because it allows for thermodynamic analysis [19], understanding the mechanism of action of micro-organisms through biodegradation, and a plethora of other properties and applications (Table 1).

Numerous experiments have been conducted on model bacterial membranes utilizing this technique, most frequently using monolayers made of phospholipid mixtures such as dimyristoyl phosphatidylglycerol (DMPG), dimyristoyl phosphoethanolamine (DMPE), and CL at various ratios [68]. As model membranes for Gram-positive membranes and Gram-negative inner membranes, Langmuir monolayers composed of pure bacterial phospholipids (PE, PG, CL) or mixtures of phospholipids have been used [16]. In a study by Perczyk et al. [69], models of soil bacterial membranes that varied in the mutual ratio of primary phospholipids were created utilizing single phospholipids, such as DMPE, DMPG, phosphatidylethanolamine (POPE), dipalmitoyl phosphoethanolamine (DPPE), dipalmitoyl phosphoglycerol (DPPG), and dioleoyl phosphatidylglycerol (DOPG), as well as their mixtures at different molar ratios. Additionally, few studies have reported the interactions of antibiotics with model bacterial membranes using *E. coli* extract, DOPC, or LPSs dissolved in a CHCl_3_ solution [70,71,72].

Another advantage of this model, as shown in these studies, is the ability to achieve varying densities and compositions of lipids at the interface in a controlled manner [40], which was made possible because of the ease of manipulation of the phospholipid composition in the monolayer compared to a bilayer (Table 1). Controlling the content of individual phospholipids in membranes is of great importance, as changes in the composition of acyl chains or headgroups affect fluidity and stability. Consequently, they influence the membrane response to environmental perturbations [40]. Therefore, the Langmuir technique can be used to provide a simple platform for manipulating phospholipid composition in a monolayer to understand the precise roles played by membrane phospholipids in bacterial physiology and stress adaptation, which has not yet been completely realized [40].

Rowlett et al. [73] exploited this property by systematically manipulating the phospholipid composition of E. coli membranes. They found that changes in CL or PE levels led to changes in the structure and composition of the cellular envelope, homeostatic pathways, and membrane biogenesis. Furthermore, phospholipid-modified strains showed disruption in surface adhesion with altered susceptibility to environmental stress. This indicates that maintaining proper membrane phospholipid composition is important for bacterial adaptation. The main drawback of this technique lies in its inherent ability to simplify the membrane system into one leaflet, which is beneficial for investigating specific interactions at the molecular level but simultaneously interferes with the accurate understanding of some membrane functions by not reflecting the complexity of a true biological membrane (Table 1).

### 4.2. Supported Lipid Bilayers (SLBs)

Supported lipid bilayers (SLBs) are robust biomimetics that are widely used as models for mammalian and bacterial membranes [16,35]. There are different types of supported lipid membranes, such as solid-supported, tethered, or polymer-cushioned lipid bilayers. All of these systems rely on either lipid-surface interactions or molecular anchors that attach lipid sheets to structural supports. These models can be further classified based on the type of support and the techniques used to produce them.

The lipids typically used for SLB formation poorly resemble those of bacterial cell membranes due to the lack of available protocols to form SLBs relying solely on mixtures of lipids relevant for bacteria such as PE and PG [23]. Few reports have been published on the formation of SLBs utilizing mixtures of PC with PE [74,75], or single component PC membranes to determine the mechanism of action of antimicrobial compounds [76].

#### 4.2.1. Vesicle Fusion to form SLBs

One popular method to form SLBs is vesicle fusion due to its simplicity [16]. The formation of small vesicles is usually performed by tip sonification or extrusion through a filter [35,52,77]. The vesicle fusion method for forming a solid-supported bilayer is composed of three main stages: vesicle adsorption, rupture, fusion, and SLB adhesion [77]. At the initial stage, small vesicles are adsorbed to the substrate surface, such as silica, glass, an oxidized layer on a silicon wafer, and mica, which are negatively charged. Because of the electrostatic repulsion between negatively charged lipids and the substrates, a divalent cation such as Ca2+ is usually added to allow the fusion of vesicles to take place, forming the SLB.

The technique of vesicle fusion has several advantages associated with the nature of the vesicles used, such as obtaining more complex and selective bilayers [40]. In addition, it results in the formation of a double layer that mimics biological membranes better than the Langmuir monolayer and a more stable membrane compared to lipid vesicles [40]. However, owing to the use of solid support, the interaction between one lipid monolayer and the support is very strong, thus impacting its physicochemical properties.

#### 4.2.2. Langmuir−Blodgett/Langmuir−Schaefer to form SLBs

As an alternative to vesicle fusion, Langmuir–Blodgett/Langmuir–Schaefer (LB/LS) deposition has been used to assemble advanced models of bacterial SLBs [16,35]. This technique deposits lipid bilayers on substrates by combining the vertical dipping Langmuir–Blodgett (LB) deposition technique with the horizontal dipping Langmuir–Schaefer (LS) technique.

A lipid monolayer is first generated by LB deposition (Figure 3D). This layer is then transferred onto a hydrophilic substrate by immersing it into a subphase and then vertically lifting the substrate using the LB deposition method, creating the lower leaflet of the bilayer [78]. The substrate is then horizontally oriented with the side coated by the first layer lying parallel to the air-water interface and slowly lowered until the entire surface is in contact with the floating monolayer, creating a tail-to-tail interaction. The substrate is then pushed through the interface to deposit the second layer [78].

#### 4.2.3. Langmuir−Blodgett/Langmuir−Schaefer to form Free “Floating” SLBs

In addition to single-SLBs, the combination of Langmuir– Blodgett/Langmuir–Schaefer deposition techniques can be used to form floating membranes or supported double bilayers [35]. These multi-bilayer configurations were developed to further minimize the loss of mobility due to lipid-surface interactions because the floating bilayer is not restrained by the solid substrates. These membranes interact via electrostatic, hydration, and van der Waals forces, which creates more room for protein integration but also affects the model's stability, creating a set of other drawbacks (Table 1). However, not all phospholipids can form stable double bilayers; for example, double bilayers of DPPE were found to be stable in the gel phase but destabilized in the fluid phase [44]. Although this method is significantly more time-consuming and requires high technical skills, the membranes produced by LB/LS are asymmetric in nature and can be used to represent an accurate model of the outer membrane of Gram-negative bacteria.

**Figure 4:**
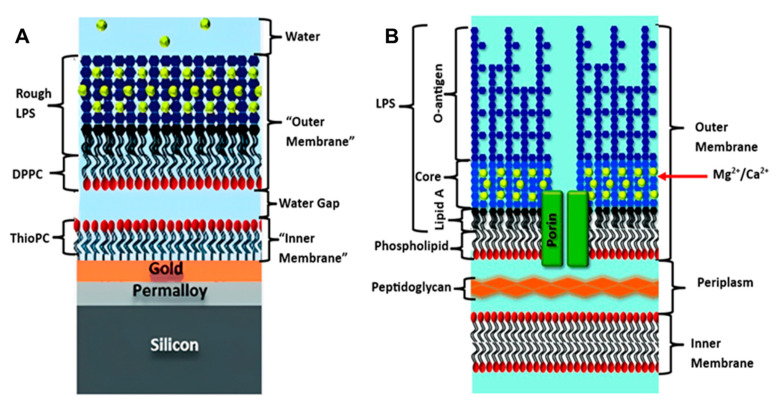
(**A**) Floating model of Gram-negative bacterial membrane, (**B**) Original Bacterial membrane structure. [reproduced with permission from Clifton, et al. [45], copyright 2015 Wiley].

To date, floating bilayers, have not been heavily explored for bacterial membrane biomimetics studies. In a hybrid floating membrane model was developed by Clifton et al. to mimic the asymmetrical outer membrane structure and dynamics of Gram-negative bacteria [45]. These hybrid systems are usually created by depositing a lipid monolayer onto a hydrophobic surface, such as gold or a polymer film coated with a self-assembled monolayer (SAM) [79,80], resulting in the formation of an asymmetric structure. However, this model had some limitations, in that the outer membrane in the real bacteria is connected to the periplasm by proteins (Figure 4B) and does not float freely, as in the model (Figure 4A). This hybrid system sheds light on the power of the floating outer membrane model for studying the biological and biophysical behaviors of bacterial membranes, which is in line with other characteristics and properties (Table 1).

### 4.3. The Architecture of Mimetic SLBs Supports

In the systems described above, the bilayer is always separated from the substrate by a thin film of water (∼10–20 Å) [81]. Although the lubricating water film maintains the long-range fluidity of the membrane [82], the space provided and the stability of the system are not sufficient, presenting many limitations. One of the main drawbacks is the restriction in the lateral diffusion of lipids, which occurs due to interactions between the lipid headgroups and the substrate. In addition, the water layer is too small to accommodate the large hydrophilic domains of integral proteins if needed for biosensing applications. Besides, the bilayer is barely physically linked to the surface; therefore, it can easily be detached from the surface [35].

**Figure 5:**
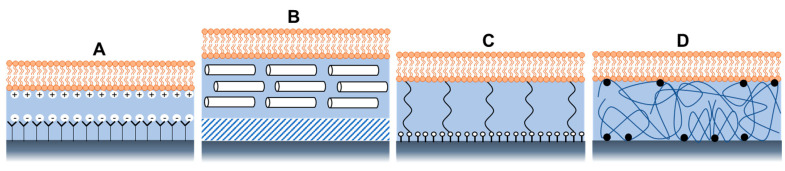
Architectures of supported lipid bilayers, using (**A**) Self-assembled monolayers with a charged head group, (**B**) soft polymer cushion, (**C**) using hydrophilic (low molecular weight) spacer molecules, and (**D**) using a hydrophilic polymer cushion.

Membrane stability and mobility depend on several factors, such as pH and salt concentration, which must be maintained within specific limits to control interactions with substrates [35]. Apart from maintaining the solution conditions, other modifications of the system architecture were explored to create various composite systems to reduce lipid-substrate interactions [35]. These composite systems were found to be more useful for improving and investigating other physicochemical properties of bacterial membranes, such as their structural and electrical properties. Some examples are shown in Figure 5, such as a bilayer supported on a SAM terminated with polar or charged head groups (Figure 5A) or on a hydrophilic soft polymer cushion (Figure 5B), such as an ultrathin layer of dextran, or cellulose, showing longer-range mobility and better membrane stability [35].

Tethered polymer cushions are another widely used model of bacterial biomimetic membranes. They are placed beneath the SLBs to lift them from the support while maintaining their structural integrity [46]. The bilayer can covalently attach to the solid support via hydrophilic tethers (Figure 5C) or polymer cushions (Figure 5D). By manipulating the charge, chemical structure, and hydrophilicity of polymers, many proteins have been successfully added with varying levels of mobility and apparent functionality using this model [48,49]. Besides, the tether design facilitates the formation of a larger aqueous region between the lipid bilayer and the substrate, which can accommodate the extra- or intracellular domains of transmembrane proteins and provide an ionic reservoir required for ion channel function. In addition, the hydrated tether region or polymer acts as a screen that “decouples” the bilayer from the substrate [35] to prevent the interactions of the lipids with the underlying solid substrate.

Biomimetic membranes based on tethered polymers are among the most successful membrane-based biosensors. Andersson et al. [83] created a model membrane that mimicked the outer membrane of Gram-negative bacteria, which was then used to investigate the structural and electrical properties concerning the influence of divalent ions and antibiotics. The structure of the model is based on a tethered monolayer fused with vesicles containing LPS molecules. Other characteristics, such as membrane diffusivity, viscoelasticity, mobility, and lipid and protein symmetry, were investigated by the Daniel group [50], who developed a model membrane of the outer membrane of Gram-negative bacteria. Changes in the membrane properties, mass, and kinetics were also investigated in the presence of antibiotics.

## 5. Bacterial Membranes in Drug Discovery and Biosensing

**Figure 6:**
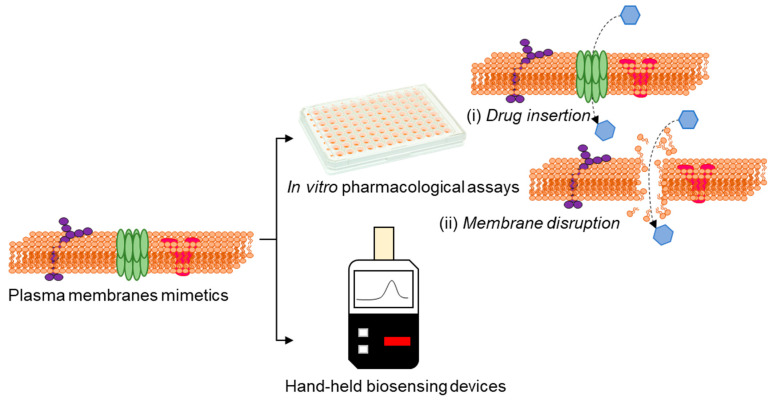
Advances in technologies using plasma membrane mimetics allows us to perform drug discovery and sensing by in vitro assays based on drug insertion (**i**) and membrane disruption (**ii**) and conceive the possibilities of real-time membrane-based hand-held biosensing devices.

Activities of bacterial membrane efflux pumps/porins and alterations in the membrane characteristics can greatly affect the antibiotics’ efficacy [84,85]. In this perspective, attempts are directed to utilize transducers that enable ionic signal transduction in a label-free manner [86], for the direct assessment of drug-membrane interactions (Figure 6), which would facilitate high-throughput/content drug testing assays and also real time sensing of membrane events.

**Figure 7:**
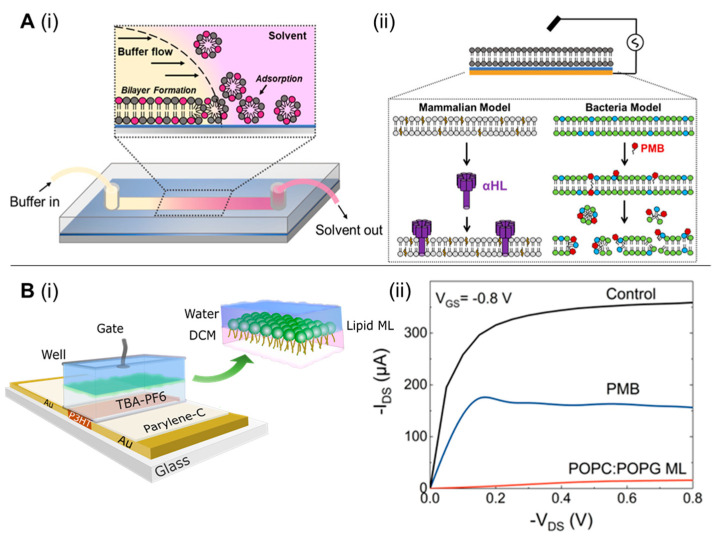
(**A**) Solvent-assisted lipid bilayer formation in a microfluidic channel (**i**); EIS configuration used in this work (**ii**). The thin blue layer represents the PEDOT:PSS film, and the gold layer represents the gold contact supporting the polymer and planar bilayer. Below the diagram are cartoon representations of the two bilayers we formed with this method and the toxin (α-HL) or compound (PMB-polymyxin B) used to disrupt them. POPC-1-palmitoyl-2-oleoyl-sn-glycero-3-phosphocholine (light gray) and cholesterol (yellow) are principal components of the mammalian bilayer, and POPE (green- 1-palmitoyl-2-oleoyl-sn-glycero-3-phosphoethanolamine) and POPG-1-palmitoyl-2-oleoyl-sn-glycero-3-phospho-(1′-rac-glycerol) (blue) are the components used for the bacterial bilayer (**ii**). [reproduced with permission from Su, et al. [90], copyright 2019 American Chemical Society]. (**B**) Schematic structure of the biphasic–electrolyte gated organic electrochemical transistor (OECT) system showing the lipid monolayer formation at the interface with the head and tail groups oriented toward the aqueous phase and organic phases, chemical components used (**i**); OECT recordings of the interaction of PMB with the bacterial model system, comparative transfer and output plots showing the change on the electrical behavior of the OECT before and after the formation of the POPC:POPG lipid ML and subsequent addition of polymyxin B (**ii**). [reproduced with permission from Pitsalidis et al. [92], copyright 2018 American Association for the Advancement of Science].

To this end, interfacing organic electronic devices with cell-membrane models have shown great promise in various applications, including ion channel activity studies [87,88,89]. Su et al. [90] devised a versatile and facile technique to generate supported lipid bilayers on a conducting polymer film surface by a solvent-assisted lipid bilayer approach. The generated bacterial membrane mimetic bioelectronic device was used to monitor the interaction of a bacterial membrane with the antibiotic polymyxin B. The technique was found to be compatible with various membrane compositions and to efficiently work in microfluidic devices (Figure 7A). The use of an organic electrochemical transistor (OECT), a highly sensitive ion-to-electron converter [91], for screening molecules that particularly disrupt/alter the permeability of a bacterial membrane has been also reported [92]. Specifically, a lipid monolayer was incorporated in a liquid-liquid interface on top of an OECT device, acting as the bacterial membrane (Figure 7B). Alterations in ionic flux caused by permeabilizing compounds were elucidated using the OECT device, indicating a bacterial membrane disruption. The role of biomembrane-based organic electrochemical transistors as rapid and low-cost biosensing platforms, followed by their advantages and limitations, has been reviewed by Lubrano et al. [93]. Recently, BM-antibiotic interaction were reported by Ghosh et al. [94] who created a surface-supported planar BM model on an optically-transparent, conducting polymer to determine the impedance of BM-antibiotic interaction using polymyxin B, bacitracin, and meropenem. This technique was also expanded to study clinically relevant ESKAPE pathogens, such as *Acinetobacter baumannii*, *Enterobacter cloacae*, and *Pseudomonas aeruginosa* [95]. Such surface-supported platforms developed from BM of clinically significant bacteria retaining BM characteristics such as fluidity, membrane proteins, and lipopolysaccharides have immense potential for screening and identifying BM-specific antibiotics [96], peptides [97], nanomaterials [98,99] or developing recombinant phages targeting BM surface components.

The natural ability of bacteria to selectively sort proteins to both the interior and exterior of the BMs is also exploited to develop engineered BM vesicles (BMVs) for biosensing and imaging applications [100]. For instance, Chen et al. [101] developed a one-pot synthesis approach to engineer *E. coli* BMVs-based multi-functional sensors for both antigen targeting and signal generation. A fusion peptide, SlyB, was used to package nanoluciferase within BMVs, and the INP-Scaf3 scaffold was used for antibody recruitment. The multifunctional BMVs could detect thrombin with a 0.5 nm detection limit. By inserting a cohesion domain, these engineered BMVs were functionalized with GFP for imaging cancer cells. Apart from using BMs as a whole for biosensing, BM components such as aquaporins, transporters, ion channels, and receptors are also explored to develop biosensors. Similar to BM mimetics for biosensing, even components of BM are used for biosensing applications; readers can refer to Novikova et al. [102] and Ryu et al. [103].

**Figure 8:**
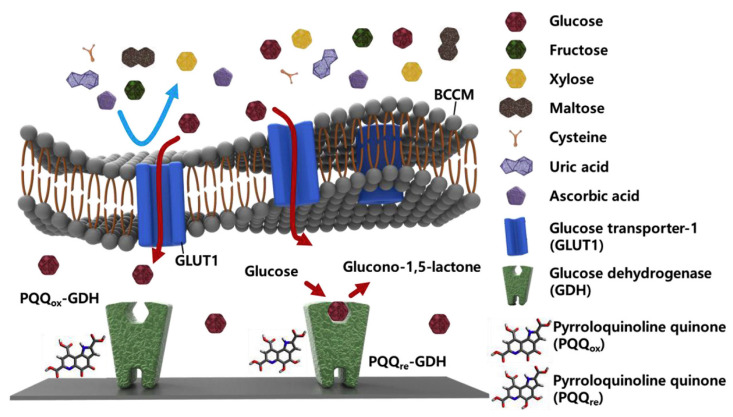
Schematic representation of enzymatic glucose sensor coated with breast cancer cell membrane (BCCM). The BCCM acts as a diffusion barrier to impede the transport of large and uncharged molecules (fructose, xylose, and maltose) and ions (cysteine, uric acid, and ascorbic acid), whereas it facilitates the active transport of glucose via glucose transporter-1 (GLUT-1). Glucose dehydrogenase (GDH) then reacts with glucose without any interference. [reproduced with permission from Kim, Kwon, Lee, Lee, and Yoon [104], copyright 2019 Elsevier].

Taking clues from animal cell membrane-based biosensing platforms, BMs could be utilized as biorecognition elements for sensing biomolarkers. For instance, breast cancer cell membrane (BCCM) extracted from MDA-MB-231 and coated onto an enzyme-deposited electrode via vesicle fusion technique were used for glucose sensing [104]. Excessive and selective uptake of α-D-glucose by the glucose transporter-1 on the BCCM allowed the specific and sensitive detection (Figure 8). The exceptional permselectivity of BCCM coated biosensor was evaluated using L-cysteine, L-ascorbic acid, D-(−)-fructose, D-(+)-xylose, D-(+)-maltose, uric acid, and human serum as various interfering biomolecules. Similarly, the selectively of glucose transporter-1 in the red blood cell membrane (RBCM) was also exploited for the detection of glucose [105]. The RBCM sensors showed good reliability at different glucose concentrations and long-term stability. 

Combining BM mimetics with photosensitive quantum dots provides an opportunity to develop photosynthetic semiconductor biohybrids (PSBs). The colloidal quantum dots in PSBs capture light to regulate biochemical processes in the biosystems such as BMs. Recently, Suri et al. [106] developed a bioelectronic system that decreases the intricacies associated with PSBs by focusing explicitly on interactions between P. aeruginosa BM, quantum dots, and pyocyanin redox mediators. Chronoamperometry, revealed that photoexcited charge transfer in this platform was driven by the reduction of pyocyanin at the surface of colloidal quantum dots followed by diffusion of reduced pyocyanin through the BM. The broad applicability of this PSBs system under a controlled environment across many bacterial species and diverse quantum dot architectures showcases the possibilities at the interface of BM mimetics and inorganic nanomaterials to develop advanced biosensing devices. 

## 6. Bacterial Membranes in Immunomodulation

### 6.1. Bacterial Membranes against Bacterial Infections

Vaccines based on BMVs have attracted significant attention in the recent past. BMVs are immunogenic spherosomes secreted by Gram-negative bacteria [107]. BMVs can modulate/suppress immune cell responses via direct interaction with the host cells. BMVs are stable, non-infectious, and genetically tractable nanoparticles (NPs) containing major immunogenic proteins of the parent bacterium with the capacity to elicit both arms of the immune system, suggesting their suitability as vaccines and adjuvants [108]. For instance, *Neisseria meningitidis*-OMVs is a licensed vaccine to control meningococcal B disease in humans [109]. Traditionally, the synthesis of *N. meningitidis*-OMVs followed the extraction of vesicles from the biomass post-detergent treatment (dOMVs). Further, genetic detoxification of *N. meningitidis* LPS allowed detergent-free extraction of OMVs (eOMVs) [110]. However, current attempts are focused on enhancing the yield of spontaneous OMVs (sOMVs), i.e., without the use of detergents or chelating agents that are required in the case of dOMVs and eOMVs, respectively [111]. On the other hand, the natural OMNs (nOMVs) produced by the disruption of bacteria through sonication/vortexing is another approach, where nOMVs have better immunogenic efficacy due to their structural/functional relatedness to the BM. Vaccination with nOMVs of *lpxL1* mutant (a variant strain of *N. meningitides* with pentacylated LPS) produced an immune response marked by complement stimulation via factor H binding and high antibody response, particularly IgG titers. Also, different routes for administering *N. meningitides* OMVs are reported; for instance, intramuscular [112], intraperitoneal [113], and subcutaneous [114].

BMVs have been also exploited to tackle antibiotic-resistant bacterial infections. Both natural and engineered BMVs isolated directly from the source of bacteria or from genetically engineered bacteria, respectively, are tested against homologous and heterologous bacterial infections [115] (Table 2).

### 6.2. Bacterial mem2branes against Viral Infections

The OMVs can serve as an attractive vaccine delivery vehicle for viral antigens because of their self-adjuvant characteristics and the amenability to be decorated with antigens. Loading of OMVs with heterologous antigens by directing protein expression onto the outer membrane or into the lumen leads to the efficient surface display of foreign protein with OMVs proteins [123]. The bacterial hemolysin ClyA is one of the best-described carrier proteins [124]. Considering the continued emergence of SARS-CoV-2 variants, considerable attention is given to efficiently displaying multivalent antigens on OMVs. For instance, Wo et al. [125] engineered probiotic-derived OMVs as vaccine carriers to present SARS-CoV-2 antigens. A bivalent antigen display platform was developed to showcase the simultaneous presentation of two different virus antigens in the lumens and on the surface of OMVs. When tested on mice, ClyA-NG06 fusion and the receptor-binding domain (RBD) of the spike protein in the OMV lumen elicited a stronger humoral response than OMVs presenting either the ClyA-NG06 fusion or RBD alone. Today, different types of bacteria are explored to generate engineered OMVs displaying SARS-CoV-2 antigens, such as *E. coli* [125], *N. meningitides* [126], and *Vibrio cholerae* [127], which could be administered via intranasal and intraperitoneal routes. This approach can be extended to tackle other viral infections in the future.

### 6.3. Bacterial Membrane-Coated Nanoparticles against Bacterial Infections

**Figure 9:**
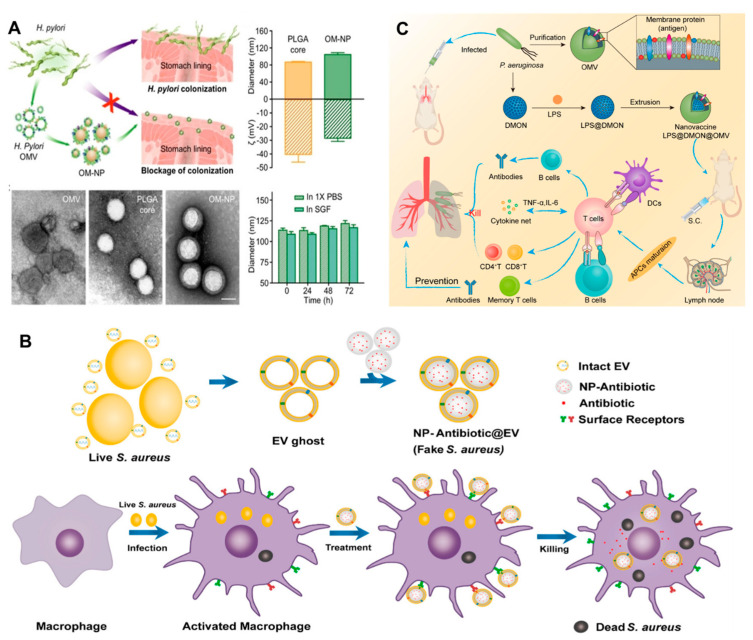
(**A**) Schematic representation of BM-PLGA [BMs coated on polymeric cores made of poly (lactic-co-glycolic acid] mediated inhibition of *H. pyroli* adhesion on the stomach lining; TEM, hydrodynamic diameter, the zeta potential of BM-PLGA and its stability in 1 X PBS and simulated gastric fluid [reproduced with the permission from Zhang et al. [136], copyright 2019 Wiley]. (**B**) Schematic representation of the preparation of PLGA@BMs, by fusing the derived membrane vesicle of the secreted by *S. aureus* over the surface of PLGA nanoparticles, exposure of a macrophage to *S. aureus* leads to phagocytosis, consequent antigen presentation, and re-exposure of the phagocyte to the same pathogen type leads to rapid identification and clearance [reproduced with permission from, Gao, et al. [138], copyright 2018 American Chemical Society]. (**C**) Schematic representation of the antipseudomonal immune regulation of LPS@DMON@OMVs encapsulated by BMVs [reproduced with permission from Wu et al. [139], copyright 2022 Elsevier].

Adequate literature has already contextualized the potential of BMVs as a clinical vaccine or for immunomodulation [107,128,129]. However, due to the low structural stability and limited homogeneity of BMVs, the immune response remains elusive [130]. In this regard, the last decade witnessed a rise in the literature on BM-coated NPs for immunomodulation against bacterial infections. BMV coating bestows stability, systemic longevity, biocompatibility, and targeting ability to the resultant BMV@NP conjugate, which is an added advantage for the tunable physio-chemical characteristics of NPs. NPs derived from different materials such as metallic, polymeric, composite, magnetic, silica, and carbon-based offer tremendous opportunities to develop a spectrum of NPs with diverse structures and functionalities. NPs are explored as carriers of poorly soluble compounds [131,132] to penetrate across biological membranes [133], targeted therapy, and theragnostic applications [134]. Gao et al. [135] collected BMVs and successfully coated them on gold NPs (AuNPs) to give BM-AuNPs with marked stability in biological buffers. The BM-AuNPs triggered the activation and maturation of dendritic cells (DCs) in the lymph nodes of vaccinated mice. Compared to the vaccination with BMVs alone, BM-AuNPs generated immune response was durable and of higher avidity; even the secretion of interferon-gamma and interleukin-17 was enhanced, but not interleukin-4, suggesting the capacity to induce Th1 and Th17 biased cell responses. This finding indicates that BM-AuNPs synthesized using a top-to-bottom approach preserved the immunogenic determinants on the BMs to trigger antibacterial immune responses and work as bionic bacteria. Similarly, *Helicobacter pyroli* BMs coated on polymeric cores made of poly (lactic-co-glycolic acid) (PLGA) using the nanoprecipitation method could bind with gastric epithelial cells [136]. BM-PLGA competed with source bacteria to bind the host cells (Figure 9A); however, this adhesion is dependent on the BM-PLGA concentration and its dosing sequence. This top-down fabrication strategy avoids the screening/identification of adhesins and holds the potential to bypass the design of agonists targeting these adhesins. Adriani et al. [137] synthesized *V. cholerae* BMVs encapsulated chitosan NPs which were coated with Eudragit. Mucosal immunization with nanovaccine provided protection against 10^8^ CFU of *V. cholerae*.

*Staphylococcus aureus* complications arise due to its ability to survive inside host phagocytes, particularly macrophages. This makes it difficult to eliminate intracellular *S. aureus* due to the limited penetration of antibiotics, thus, necessitating intracellular delivery of antibiotics. To tackle the issue, Gao et al. [138] implemented a “Trojan horse” strategy, i.e., killing the real using fake bacteria. PLGA NPs coated with secreted extracellular vesicles from BMs of *S. aureus* (NP@EV) were designed to target as active drug carriers (Figure 9B). NP@EV showed higher efficacy of internalization by *S. aureus*-infected macrophages than the naïve counterparts. Intravenous injection of NP@EV resulted in accumulation within four major organs bearing *S. aureus* infection. This observation suggests that BMV coating bestows the NPs with targeting ability. Such an actively-targeting system has the potential to promote clinical success for intracellular pathogen-associated infections. Along similar lines, Chen et al. [140] demonstrated that *S. aureus* BMV-coated magnetic mesoporous silica loaded with indocyanine green (EV/ICG/MSNs) could be used as multi-antigenic vaccines with the capacity to modulate antigen-presenting pathway in DCs for stimulating an immune response. Laser irradiation of EV/ICG/MSNs promoted DC maturation, followed by activation of proteasome-dependent antigen presentation by facilitating endolysosomal escape. Immunization with EV/ICG/MSNs coupled to laser irradiation in vivo stimulated CD8^+^T cell response. Skin experiments showed that EV/ICG/MSNs could prevent and treat superficial infections by inhibiting *the* invasiveness of *S. aureus*. Another study [130] explored the potential of *Klebsiella pneumoniae* BMVs by reinforcing them internally with size-controlled and stable bovine serum albumin NPs (BN-OMVs). The synthesized BN-OMVs exhibited core-shell morphology, and the size was around 100 nm. Immunization with BN-OMVs by subcutaneous injection resulted in a significantly high antibody titer and survival of immunized mice infected with a lethal dose of *K. pneumoniae*. Recently, Wu et al. [139] developed a nanovaccine encapsulated in *P. aeruginosa* BMVs to enhance immunotherapy against bacterial pneumonia. Nanovaccine (LPS@DMON@OMVs) synthesized using lipopolysaccharide, dendritic mesoporous organosilica NPs using non-replicable BMVs as antigen and LPS as adjuvants triggered activation and maturation of DCs. Antibody titer stimulated by LPS@DMON@OMVs was 180 times higher than that of free BMVs. It also induced the generation of cytokines and memory T cells (Figure 9C).

### 6.4. Bacterial Membranes to Deliver Antibiotics

**Figure 10:**
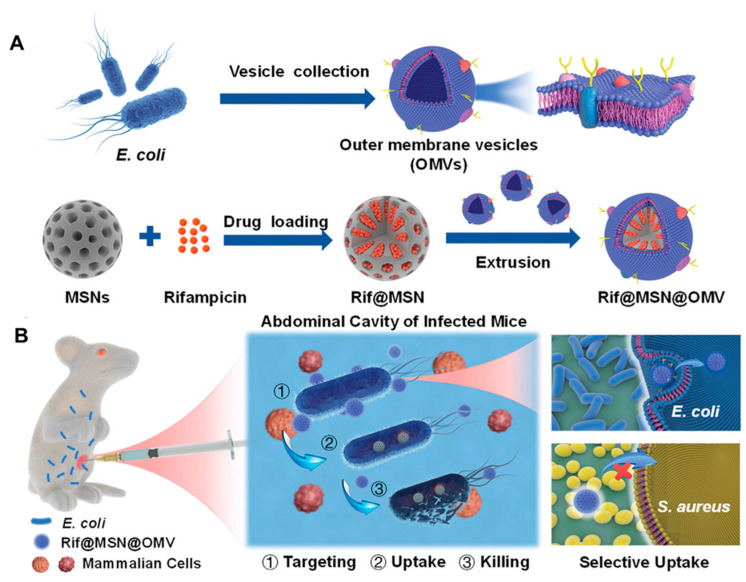
(**A**) Schematic representation of the Rifampicin loaded mesoporous silica nanoparticles coated with bacterial membrane vesiclesand its application in treating bacterial infections in the (**B**) mouse model. [reproduced with permission from Wu et al. [141], copyright 2021 Wiley].

Apart from immunomodulation and intracellular targeting, BMV-NPs and bare BMVs are capable of delivering antibiotics in a target-specific manner. For instance, to enhance the efficacy of conventional antibiotics, Wu et al. [141] developed a biomimetic nano-delivery system consisting of BMVs derived from *E. coli* and rifampicin-loaded mesoporous silica NPs (Figure 10A). The biomimetic coating of BMV enabled the selective uptake of the NPs by *E. coli* but not *S. aureus* due to homotypic targeting (Figure 10B). This system, completely eradicated the bacteria at the concentration of 4 µg/mL compared to free rifampicin while elevating the survival rate of mice and maintaining good biocompatibility in both in vitro and in vivo conditions. Similarly, Huang et al. [142] exploited the ability of *A. baumannii* to efflux antibiotics into periplasmic spaces, followed by packing in BMVs for developing nanoantibiotics. The novel nanoantibiotics consisted of BMVs loaded with antibiotics, which showed excellent bactericidal effects both in vitro and in vivo. Nanoantibiotics were biocompatible and demonstrated effective protection against enteric *E. coli* infection with oral administration.

## 7. Bacterial Membranes in Cancer Immunotherapy

The use of BMs in cancer immunotherapy is still an under-explored area but has gained attention in recent years. A critical limitation of photothermal therapy (PTT) hinders the implementation of systemic anticancer therapies owing to unsatisfactory photothermal conversion efficacy, insufficient tumor accumulation, and lack of anticancer immunity of PTT agents. In this regard, Qin, et al. [143] exploited BMVs derived from *Escherichia coli* Nissle 1917 as the nanoreactors to fabricate biomimetic copper sulfide NPs (CuS-BMVs) to perform systemic photothermo-immunotherapy. The CuS-BMVs exhibited photostability, tumor-targeting ability, and excellent photothermal conversion efficacy. The CuS-BMVs cause hyperthermia as a result of second near-infrared irradiation at the tumor site. Also, strong immunogenic tumor cell death, DCs maturation, and CD8^+^ T cell activation were observed. Further, CuS-BMVs acted as immune-adjuvants and repolarized M2-like tumor-associated macrophages into M1-like phenotypes for reshaping the immune-suppressive tumor microenvironment. Neoantigens that are specific to an individual’s cancer are major tumor determinants recognized by T cells.

Limited recognition of neoantigens in immunologically cold tumors results in a lack of antitumor immunoregulation, making it is as a clinical challenge to target such tumors, which are unresponsive to most immunotherapies. Further, radiation therapies alone barely result in a systemic antitumor response. To tackle this, Patel et al. [144] developed a multifunctional BM-NPs composed of immunomodulating PC7A/CpG polyplex core encapsulated with BM and imide group to increase antigen retrieval. The BM-NPs could bind neoantigens following radiation treatment and increase their uptake and cross-presentation to dendritic and T cells, respectively. Treatment of mice with syngeneic melanoma and neuroblastoma with BM-NPs and radiation therapy resulted in the activation of antitumor immune regulation.

**Figure 11:**
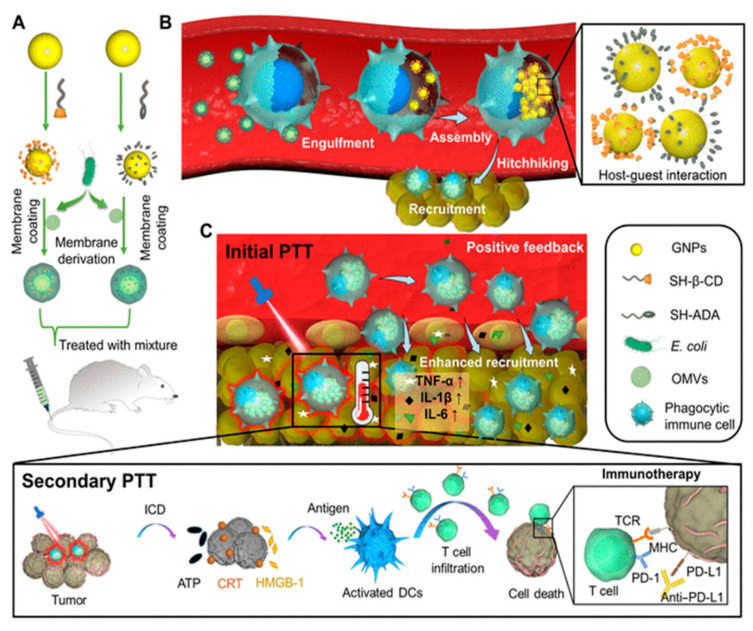
(**A**) *E. coli* BM coated on β-cyclodextrin-adamantane to prepared BM-NPs. (**B**) Selective phagocytosis of BM-NPs (Bacterial membrane coated nanoparticles) followed by degradation of coated BM and subsequent intracellular aggregation of NPs by host-guest binding of β-cyclodextrin-adamantane. (**C**) Primary photothermal therapy of tumor trigger inflammatory signals and provides a positive feedback to recruit immune cells carrying NP aggregates, secondary photothermal therapy induces significantly enhanced antitumor immune response. [reproduced with permission from Gao et al. [145], copyright 2022 AAAS].

Animal cell-based drug carriers prepared in vitro may induce negative physiological function of cells and cause possible immune rejection when used in different individuals. Further, the immune suppressive tumor microenvironment limits immune cell-mediated delivery. In this regard, Gao et al. [145] developed *E. coli* BM-coated NPs (gold core modified with β-cyclodextrin and adamantane), which, when injected intravenously, hitchhike circulating phagocytic immune cells by selective phagocytosis (Figure 11A). This leads to intracellular degradation of BMVs and supramolecular self-assembly of NPs driven via β-cyclodextrin-adamantane host-guest binding (Figure 11B). This results in the aggregation of NPs (with photothermal effect), which were previously dispersed (without photothermal effect), leading to a significant reduction in the leakage of NPs from the immune cells. Further, the inflammatory tropism generated by primary photothermal treatment of tumor results in the movement of immune cells carrying NPs toward tumor tissue in vivo. Positive feedback recruits more immune cells (including carriers of intracellular NPs aggregates), leading to enhanced tumor accumulation of NPs for secondary photothermal therapy (Figure 11C). The immune cell hitchhiking strategy offers new insight into the use of BM mimetics for minimizing the loss of nanomedicine and improving the efficacy of photothermal therapy.

## 8. Augmenting the Functionalities of Nanomaterials

The amenability of nanomaterials towards fabrication and availability in different dimensionalities make them versatile tools for drug sensing, discovery, and delivery applications. Metallic, metal-oxides, magnetic, silica, carbon-based, polymeric (both synthetic and biodegradable), and bio-inspired nanomaterials are being reported for either or all such applications. For instance, gold NPs could help in the preservation of complex biological characteristics of coated BMs (BM-NPs) and better mimic antigen presentation by bacteria than just BMs. This combines the merits of two distinct materials, thus increasing the expectation to generate strong antibacterial or immunogenic responses [135]. The properties of nanomaterials could influence the behavior of the coated/encapsulated materials [146]. For instance, nanomaterials with inherent toxicity or poor biocompatibility may restrict the desired application. Therefore, nanomaterials that are predominantly biocompatible such as gold, could be preferred for in vivo application [135,147]. In another way, the biocompatibility of nanomaterials can be enhanced or modulated by coating them with either biomolecules and biocompatible materials such as proteins/amino-acids/metabolites [148,149], and biodegradable polymers like chitosan, respectively [131]. Similarly, the inherent characteristics of some NPs could be exploited to encapsulate both hydrophilic and hydrophobic drugs/fluorescent molecules prior to BM coating for targeted delivery and biosensing in bacterial infections, cancers, and immunomodulation. For instance, as an effective approach, hydrophilic–hydrophobic chemotherapeutic drug pairs (paclitaxel and doxorubicin) were co-encapsulated into magnetic O-carboxymethyl-chitosan NPs [150]. Further, to endow these NPs for programmed delivery, they were camouflaged with an Arg-Gly-Asp anchored erythrocyte membrane. Compared to the contemporary approach of polyethylene glycol coating, this biomimetic strategy demonstrated better circulation time, improved tumor accumulation, facilitated tumor uptake, and tuned the intracellular fate of NPs. For more details, readers can follow the existing literature by Fang et al. [151], Kroll et al. [152], and Zou et al. [153] on the cell membrane coating nanotechnology for application in drug delivery, detoxification, and immunomodulation. 

## 9. Augmenting the Functionalities of Bacterial Membranes

**Figure 12:**
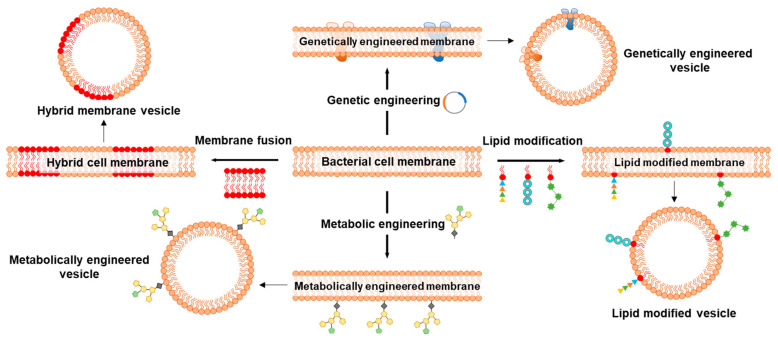
Schematics representing important strategies to modify bacterial cell membranes for application in biosensing, disease prevention, and treatment. Membrane fusion approach to producing hybrid membranes to combine the properties of two different cell types. Genetic engineering for the expression of transmembrane channels, receptors, porins, and efflux pumps that are otherwise difficult to incorporate using traditional techniques. Lipid modification strategy to enhance the affinity of cell membrane lipids to anchor ligands onto the NPs. Metabolic engineering to modify cell surface glycans that can be studied for their participation in conjugation and immunogenic responses.

Since the first report on repurposing BMs to coat NPs, a wide range of BM-NP systems have been developed and exploited for immunomodulation and cancer therapy; however, not much has happened in the field of biosensing using BMs. BM coating of electrodes for sensing or functionalization of NPs for immunomodulation and cancer therapy provides a facile strategy to recapitulate the bio-interface characteristics of the bacterial cell membrane. Functionalization of electrodes and NPs by direct use of BMs circumvents the often lengthy, expertise-, cost- and time-driven process of developing artificial ligands [154]. However, certain characteristics of BMs may limit their applicability. BMs can often possess undesirable surface markers for a given application, or those essential might not be present in sufficient quantity/quality or ratio. Therefore, different strategies, such as membrane fusion, genetic and metabolic engineering followed by lipid modifications, are adopted to improve the application and overcome undesirable characteristics of BMs (Figure 12).

Membrane fusion is an approach where cytoplasmic membranes of two distinct cell types are fused to form hybrid membranes/vesicles, which are then coated on NPs. Hybrid membranes synthesized by the fusion of BM and eukaryotic cell membranes, such as from cancer cells, are increasingly explored for their enhanced abilities [155]. Cancer cell membranes are exploited as tumor vaccine candidates due to the abundance of cancer-related antigens and are emerging contenders for homologous targeting [156]. Cancer cell membranes inherit all the membrane antigens and neoantigens, which are explored for personalized therapy; however, the membrane-associated antigens alone are often low-immunogenic, making it difficult to trigger a systemic immune response. Besides being carriers of antigens to stimulate the immunogenicity of low-immunogenic antigens [157], BMs are also reported to potentiate antitumor innate immunity for immunotherapy [158]. As aggressivity and maintenance of cytotoxic T cells is an attribute of innate immunity, hybrid membranes consisting of BMs can overcome the limited low-immunogenicity with cancer cell membranes. Numerous hybrid vesicle systems are designed and explored to stimulate innate and adaptive immunity for personalized tumor therapy. Table 3 showcases some examples of hybrid membrane vesicles alone or coated onto NPs.

Genetic engineering is a powerful strategy to modify cell membrane characteristics, particularly proteins, and has been employed for the generation of BMVs with enhanced functionalities [154]. A major advantage of this approach is the direct manipulation of transmembrane proteins, which is often an easier task compared to NP functionalization with individual BM components. Readers that are interested in understanding the know-how of genetic engineering to develop BMV carriers as vaccines and tumor-modulating agents are directed to the literature review [115]. Owing to the well-established protocols of bacterial genome editing as well as plasmid transformation to express transgene(s), it is easy to manipulate the BMs and ensure the stability of transgene as well as its protein products. Genetic engineering is a potent tool to modulate the BM surface protein expression, enabling major alterations to the structural and functional properties, including modifications to cellular-/organ-level tropism. For instance, the viral protein hemagglutinin expressed on the BM of the bacteria increased the capacity of resulting BMV-NPs to escape endosomes after cellular capture [161]. The virus-mimicking potential bestowed on BMs via genetic engineering resulted in enhanced delivery of mRNA payload to the target cell cytoplasm. Huang et al. [162] isolated BMVs from a genetically engineered *E. coli* capable of secreting BMVs containing luciferase with Z-domain (together called as NanoLuc). The multifunctional NanoLuc emitted strong blue luminescence at 460 nm after interacting with furimazine. This phenomenon of NanoLuc was further exploited for noninvasive in vivo bioimaging.

Lipid modification is another strategy to potentiate the characteristics of lipid-bilayer for wider application. For instance, though not in actual BM, the PEGylated lipid-bilayer was modified with folic acid to reduce the efficiency, and side effects of mesoporous silica NPs (MSNs) coated with a PEGylated membrane [163]. Folic acid modification rendered biological adhesion properties to the lipid bilayer-coated MSNs, which also increased their uptake by NB_4_ cells. The folic acid functionalization resulted in the effective targeting of tumors in nude mice. Pichler and Emmerstorfer-Augustin [164] summarized and highlighted the possibilities of engineering the membrane lipid for pharmaceutical interventions.

Metabolic engineering, where the source bacteria is cultured with modified sugars, followed by their display via surface glycans, is another approach to altering the BMs [154]. Glycoengineering has emerged as an interesting strategy to elicit the immune response against bacterial infections and cancer. For instance, Price et al. [165] used glycoengineered OMVs isolated from *E. coli* as vaccines to enhance antibodies and effectively combat infection by mouse *S. pneumoniae* and *Campylobacter jejuni*, confirming the versatility of this strategy. Valguarnera and Feldman [166] provide a detailed method to design and prepare glycoengineered OMVs against the prominent pathogen *E. coli* as a concept. One of the best examples is the conjugate vaccine against *Haemophilus influenzae* type b, which practically eliminated the disease caused by this bacterium in vast parts of the world [165].

A new approach is to use a cell-free expression system for the co-translational integration of membrane proteins into BMs. Manzer et al. [167] employed a cell-free expression method to demonstrate tow routes to integrate a channel protein (large conductance mechanosensitive channel) fused to green fluorescent protein into a hybrid supported lipid bilayer. The lipid bilayers were assembled either by cotranslational integration of channel protein into hybrid vesicles, followed by fusing these proteoliposomes to generate hybrid lipid bilayers, or generation of hybrid lipid bilayers and subsequently the cell-free synthesis followed by insertion of channel protein directly into the bilayer. This newly reported facile strategy can accelerate the research focused on understanding the role of several proteins for biotechnological applications, including biosensing and drug screening. Of note, an integrated database of prokaryotic and eukaryotic extracellular vesicles, EVpedia (http://evpedia.info, accessed on 15 January 2023), could be referred to as a fundamental repository to advance BM studies, which would help to understand the structure/function of BMVs and realize novel applications [168]. It provides an array of tools to search and browse vesicular components, with literature on extracellular vesicles.

## 10. Conclusions

Several strategies are being explored and developed in the quest to realize the full potential of BMs. As the fields of BM mimetics, bioelectronics and nanotechnology evolve, we can get a deeper understanding of the interacting structural and functional elements of BMs, which will proportionally increase their applicability. Further, BM mimetic platforms such as BM-on-chip and BM-NPs can accelerate the speed and accuracy of drug discovery/delivery and personalized medication. This interdisciplinary strategy provides unprecedented opportunities to harness the untapped potential of native BMs as well as engineer them to render multifunctionality and create more realistic models. It is essential to perform biophysical research on emerging antimicrobial agents using BM mimetics to investigate the actual interactions between the antibiotics and BMs. Similarly, novel and more biocompatible NPs and transducers can be explored for BM mimetic research for improved efficiency and readouts. Further, advanced characterization systems should be implemented to establish and realize near-natural BMs for such mimetic platforms. Overall, BM mimetics have huge prospects in the pharmaceutical and biomedical industry due to the anticipated biosafety and cost-efficiency, both for drug discovery and targeted therapies.

## Figures and Tables

**Table 2:** Natural and engineered bacterial membrane vesicles that have be used as vaccines against some of the deadliest homologous or heterologous antibiotic-resistant bacterial infections.

Gram Type	Bacteria Source	Natural/Engineered BMVs	Target Pathogen/Disease	Reference
*Positive*	*S. pneumoniae*	Natural BMVs	*S. pneumoniae* ST8	[116]
	MRSA	Natural BMVs	*S. pneumoniae* lethal sepsis	[117]
*Negative*	*K. pneumoniae*	Natural BMVs	*K. pneumoniae*	[118]
	*V. cholerae*	Natural BMVs	*V. cholerae* infection	[119]
	*P. aeruginosa*	Natural BMVs	*P. aeruginosa*	[120]
	*A. baumannii*	Natural BMVs	Pan drug resistant *A. baumannii*	[121]
	*E. coli*	Engineered BMVs with S. aureus HlaH35 L, SpAKKAA, FhuD2, Csa1A, and LukE	*S. aureus*	[122]

**Table 3:** Examples of hybrid membrane systems consisting bacterial and animal cell membranes.

Bacteria	Animal Cell Source	Nanoparticle	Application	Reference
*E. coli*	4T1 tumor	N/A	Personalized immunotherapy	[155]
*E. coli* DH5α	B16-F10	Hollow polydopamine	Photothermal therapy	[159]
*E. coli*	4T1 tumor	PLGA	Personalized immunotherapy	[160]

## Data Availability

Not applicable.

## References

[1] WHO (2017). World Health Organization Publishes List of Bacteria for Which New Antibiotics Are Urgently Needed.

[2] Arya S.S., Sharma M.M., Das R.K., Rookes J., Cahill D., Lenka S.K. (2019). Vanillin mediated green synthesis and application of gold nanoparticles for reversal of antimicrobial resistance in Pseudomonas aeruginosa clinical isolates. Heliyon.

[3] Oroojalian F., Beygi M., Baradaran B., Mokhtarzadeh A., Shahbazi M.A. (2021). Immune cell Membrane-Coated biomimetic nanoparticles for targeted cancer therapy. Small.

[4] Nie D., Dai Z., Li J., Yang Y., Xi Z., Wang J., Zhang W., Qian K., Guo S., Zhu C. (2019). Cancer-cell-membrane-coated nanoparticles with a yolk–shell structure augment cancer chemotherapy. Nano Lett..

[5] Xue X., Liu H., Wang S., Hu Y., Huang B., Li M., Gao J., Wang X., Su J. (2022). Neutrophil-erythrocyte hybrid membrane-coated hollow copper sulfide nanoparticles for targeted and photothermal/anti-inflammatory therapy of osteoarthritis. Compos. Part B Eng..

[6] He Z., Zhang Y., Feng N. (2020). Cell membrane-coated nanosized active targeted drug delivery systems homing to tumor cells: A review. Mater. Sci. Eng. C.

[7] Silhavy T.J., Kahne D., Walker S. (2010). The bacterial cell envelope. Cold Spring Harb. Perspect. Biol..

[8] Vollmer W., Holtje J.-V. (2004). The architecture of the murein (peptidoglycan) in Gram-negative bacteria: Vertical scaffold or horizontal layer (s)?. J. Bacteriol..

[9] Malanovic N., Lohner K. (2016). Gram-positive bacterial cell envelopes: The impact on the activity of antimicrobial peptides. Biochim. Et Biophys. Acta (BBA)-Biomembr..

[10] Le Brun A.P., Clifton L.A., Halbert C.E., Lin B., Meron M., Holden P.J., Lakey J.H., Holt S.A. (2013). Structural characterization of a model Gram-negative bacterial surface using lipopolysaccharides from rough strains of Escherichia coli. Biomacromolecules.

[11] Paracini N., Schneck E., Imberty A., Micciulla S. (2022). Lipopolysaccharides at the interface. Adv. Colloid Interface Sci..

[12] Cian M.B., Giordano N.P., Mettlach J.A., Minor K.E., Dalebroux Z.D. (2020). Separation of the cell envelope for Gram-negative Bacteria into inner and outer membrane fractions with technical adjustments for Acinetobacter baumannii. JoVE (J. Vis. Exp.).

[13] Wang X., Quinn P.J. (2010). Endotoxins: Lipopolysaccharides of Gram-negative bacteria. Endotoxins: Structure, Function and Recognition.

[14] Needham B.D., Trent M.S. (2013). Fortifying the barrier: The impact of lipid A remodelling on bacterial pathogenesis. Nat. Rev. Microbiol..

[15] Breijyeh Z., Jubeh B., Karaman R. (2020). Resistance of Gram-negative bacteria to current antibacterial agents and approaches to resolve it. Molecules.

[16] Li S., Ren R., Lyu L., Song J., Wang Y., Lin T.-W., Brun A.L., Hsu H.-Y., Shen H.-H. (2022). Solid and Liquid Surface-Supported Bacterial Membrane Mimetics as a Platform for the Functional and Structural Studies of Antimicrobials. Membranes.

[17] Strahl H., Errington J. (2017). Bacterial membranes: Structure, domains, and function. Annu. Rev. Microbiol..

[18] May K.L., Grabowicz M. (2018). The bacterial outer membrane is an evolving antibiotic barrier. Proc. Natl. Acad. Sci. USA.

[19] Eeman M., Deleu M. (2010). From biological membranes to biomimetic model membranes. Biotechnol. Agron. Société Environ..

[20] Maget-Dana R. (1999). The monolayer technique: A potent tool for studying the interfacial properties of antimicrobial and membrane-lytic peptides and their interactions with lipid membranes. Biochim. Biophys. Acta (BBA)-Biomembr..

[21] Perczyk P., Broniatowski M. (2021). Simultaneous action of microbial phospholipase C and lipase on model bacterial membranes–Modeling the processes crucial for bioaugmentation. Biochim. Biophys. Acta (BBA)-Biomembr..

[22] Swana K.W., Camesano T.A., Nagarajan R. (2022). Formation of a Fully Anionic Supported Lipid Bilayer to Model Bacterial Inner Membrane for QCM-D Studies. Membranes.

[23] Lind T.K., Skoda M.W., Cárdenas M. (2019). Formation and characterization of supported lipid bilayers composed of phosphatidylethanolamine and phosphatidylglycerol by vesicle fusion, a simple but relevant model for bacterial membranes. ACS Omega.

[24] Furusato T., Horie F., Matsubayashi H.T., Amikura K., Kuruma Y., Ueda T. (2018). De novo synthesis of basal bacterial cell division proteins FtsZ, FtsA, and ZipA inside giant vesicles. ACS Synth. Biol..

[25] Tuerkova A., Kabelka I., Králová T., Sukeník L., Pokorná Š., Hof M., Vácha R. (2020). Effect of helical kink in antimicrobial peptides on membrane pore formation. Elife.

[26] Kuehn M.J., Kesty N.C. (2005). Bacterial outer membrane vesicles and the host–pathogen interaction. Genes Dev..

[27] Schooling S.R., Beveridge T.J. (2006). Membrane vesicles: An overlooked component of the matrices of biofilms. J. Bacteriol..

[28] Renelli M. (2003). DNA-Containing Membrane Vesicles of Pseudomonas aeruginosa PAO1 and Their Genetic Transformation Potential.

[29] Domingues S., Nielsen K.M. (2017). Membrane vesicles and horizontal gene transfer in prokaryotes. Curr. Opin. Microbiol..

[30] Schwechheimer C., Kuehn M.J. (2015). Outer-membrane vesicles from Gram-negative bacteria: Biogenesis and functions. Nat. Rev. Microbiol..

[31] Brown L., Wolf J.M., Prados-Rosales R., Casadevall A. (2015). Through the wall: Extracellular vesicles in Gram-positive bacteria, mycobacteria and fungi. Nat. Rev. Microbiol..

[32] Turnbull L., Toyofuku M., Hynen A.L., Kurosawa M., Pessi G., Petty N.K., Osvath S.R., Cárcamo-Oyarce G., Gloag E.S., Shimoni R. (2016). Explosive cell lysis as a mechanism for the biogenesis of bacterial membrane vesicles and biofilms. Nat. Commun..

[33] Remis J.P., Wei D., Gorur A., Zemla M., Haraga J., Allen S., Witkowska H.E., Costerton J.W., Berleman J.E., Auer M. (2014). Bacterial social networks: Structure and composition of M yxococcus xanthus outer membrane vesicle chains. Environ. Microbiol..

[34] Prez-Cruz C., Carri n O., Delgado L., Martinez G., Lpez-Iglesias C., Mercade E. (2013). New type of outer membrane vesicle produced by the Gram-negative bacterium Shewanella vesiculosa M7T: Implications for DNA content. Appl. Environ. Microbiol..

[35] Ferrari M., Martin D.K. (2007). Nanobiotechnology of biomimetic membranes.

[36] Lu Y., Allegri G., Huskens J. (2022). Vesicle-based artificial cells: Materials, construction methods and applications. Mater. Horiz..

[37] Fazal S., Lee R. (2021). Biomimetic Bacterial Membrane Vesicles for Drug Delivery Applications. Pharmaceutics.

[38] Lombardo D., Kiselev M.A. (2022). Methods of Liposomes Preparation: Formation and Control Factors of Versatile Nanocarriers for Biomedical and Nanomedicine Application. Pharmaceutics.

[39] Rukavina Z., Vanić Ž. (2016). Current trends in development of liposomes for targeting bacterial biofilms. Pharmaceutics.

[40] Rojewska M., Smułek W., Kaczorek E., Prochaska K. (2021). Langmuir Monolayer Techniques for the Investigation of Model Bacterial Membranes and Antibiotic Biodegradation Mechanisms. Membranes.

[41] Aisenbrey C., Bechinger B. (2014). Molecular packing of amphipathic peptides on the surface of lipid membranes. Langmuir.

[42] Baoukina S., Marrink S.J., Tieleman D.P. (2009). Structure and dynamics of lipid monolayers: Theory and applications. Biomembrane Frontiers.

[43] Cavassin P., Pappa A.M., Pitsalidis C., FP Barbosa H., Colucci R., Saez J., Tuchman Y., Salleo A., Faria G.C., Owens R.M. (2020). Organic transistors incorporating lipid monolayers for drug interaction studies. Adv. Mater. Technol..

[44] Stidder B., Fragneto G., Roser S.J. (2007). Structure and stability of DPPE planar bilayers. Soft Matter.

[45] Clifton L.A., Holt S.A., Hughes A.V., Daulton E.L., Arunmanee W., Heinrich F., Khalid S., Jefferies D., Charlton T.R., Webster J.R. (2015). An accurate in vitro model of the E. coli envelope. Angew. Chem. Int. Ed..

[46] Liu H.-Y., Chen W.-L., Ober C.K., Daniel S. (2018). Biologically complex planar cell plasma membranes supported on polyelectrolyte cushions enhance transmembrane protein mobility and retain native orientation. Langmuir.

[47] Andersson J., Köper I., Knoll W. (2018). Tethered membrane architectures—Design and applications. Front. Mater..

[48] Richards M.J., Hsia C.-Y., Singh R.R., Haider H., Kumpf J., Kawate T., Daniel S. (2016). Membrane protein mobility and orientation preserved in supported bilayers created directly from cell plasma membrane blebs. Langmuir.

[49] Renner L., Pompe T., Lemaitre R., Drechsel D., Werner C. (2010). Controlled enhancement of transmembrane enzyme activity in polymer cushioned supported bilayer membranes. Soft Matter.

[50] Hsia C.-Y., Chen L., Singh R.R., DeLisa M.P., Daniel S. (2016). A molecularly complete planar bacterial outer membrane platform. Sci. Rep..

[51] Toyofuku M., Nomura N., Eberl L. (2019). Types and origins of bacterial membrane vesicles. Nat. Rev. Microbiol..

[52] Klimentová J., Stulík J. (2015). Methods of isolation and purification of outer membrane vesicles from Gram-negative bacteria. Microbiol. Res..

[53] Sonntag I., Schwarz H., Hirota Y., Henning U. (1978). Cell envelope and shape of Escherichia coli: Multiple mutants missing the outer membrane lipoprotein and other major outer membrane proteins. J. Bacteriol..

[54] Schwechheimer C., Kulp A., Kuehn M.J. (2014). Modulation of bacterial outer membrane vesicle production by envelope structure and content. BMC Microbiol..

[55] Schwechheimer C., Kuehn M.J. (2013). Synthetic effect between envelope stress and lack of outer membrane vesicle production in Escherichia coli. J. Bacteriol..

[56] McBroom A.J., Kuehn M.J. (2007). Release of outer membrane vesicles by Gram-negative bacteria is a novel envelope stress response. Mol. Microbiol..

[57] Bernadac A., Gavioli M., Lazzaroni J.-C., Raina S., Lloubès R. (1998). Escherichia coli tol-pal mutants form outer membrane vesicles. J. Bacteriol..

[58] Toyofuku M., Cárcamo-Oyarce G., Yamamoto T., Eisenstein F., Hsiao C.-C., Kurosawa M., Gademann K., Pilhofer M., Nomura N., Eberl L. (2017). Prophage-triggered membrane vesicle formation through peptidoglycan damage in Bacillus subtilis. Nat. Commun..

[59] Wei S., Jiao D., Xing W. (2022). A rapid method for isolation of bacterial extracellular vesicles from culture media using epsilon-poly-L–lysine that enables immunological function research. Front. Immunol..

[60] Alves N.J., Turner K.B., DiVito K.A., Daniele M.A., Walper S.A. (2017). Affinity purification of bacterial outer membrane vesicles (OMVs) utilizing a His-tag mutant. Res. Microbiol..

[61] Huang W., Meng L., Chen Y., Dong Z., Peng Q. (2022). Bacterial outer membrane vesicles as potential biological nanomaterials for antibacterial therapy. Acta Biomater..

[62] Siontorou C.G., Nikoleli G.-P., Nikolelis D.P., Karapetis S.K. (2017). Artificial lipid membranes: Past, present, and future. Membranes.

[63] Shashi K., Satinder K., Bharat P. (2012). A complete review on: Liposomes. Int. Res. J. Pharm..

[64] Zhang H. (2017). Thin-film hydration followed by extrusion method for liposome preparation. Liposomes.

[65] Ollivon M., Lesieur S., Grabielle-Madelmont C., Paternostre M.t. (2000). Vesicle reconstitution from lipid–detergent mixed micelles. Biochim. Biophys. Acta (BBA)-Biomembr..

[66] Drost M., Diamanti E., Fuhrmann K., Goes A., Shams A., Haupenthal J., Koch M., Hirsch A.K., Fuhrmann G. (2021). Bacteriomimetic Liposomes Improve Antibiotic Activity of a Novel Energy-Coupling Factor Transporter Inhibitor. Pharmaceutics.

[67] Hussain S.A., Dey B., Bhattacharjee D., Mehta N. (2018). Unique supramolecular assembly through Langmuir–Blodgett (LB) technique. Heliyon.

[68] Ciumac D., Gong H., Campbell R.A., Campana M., Xu H., Lu J.R. (2021). Structural elucidation upon binding of antimicrobial peptides into binary mixed lipid monolayers mimicking bacterial membranes. J. Colloid Interface Sci..

[69] Perczyk P., Wójcik A., Hachlica N., Wydro P., Broniatowski M. (2020). The composition of phospholipid model bacterial membranes determines their endurance to secretory phospholipase A2 attack–The role of cardiolipin. Biochim. Biophys. Acta (BBA)-Biomembr..

[70] Sandrino B., De Oliveira J., Nobre T., Appelt P., Gupta A., De Araujo M., Rotello V., Oliveira O. (2017). Challenges in application of langmuir monolayer studies to determine the mechanisms of bactericidal activity of ruthenium complexes. Langmuir.

[71] Moreira L.G., Almeida A.M., Nield T., Camacho S.A., Aoki P.H. (2021). Modulating photochemical reactions in Langmuir monolayers of Escherichia coli lipid extract with the binding mechanisms of eosin decyl ester and toluidine blue-O photosensitizers. J. Photochem. Photobiol. B Biol..

[72] Vandera K.-K.A., Picconi P., Valero M., González-Gaitano G., Woods A., Zain N.M.M., Bruce K.D., Clifton L.A., Skoda M.W., Rahman K.M. (2020). Antibiotic-in-cyclodextrin-in-liposomes: Formulation development and interactions with model bacterial membranes. Mol. Pharm..

[73] Rowlett V.W., Mallampalli V.K., Karlstaedt A., Dowhan W., Taegtmeyer H., Margolin W., Vitrac H. (2017). Impact of membrane phospholipid alterations in Escherichia coli on cellular function and bacterial stress adaptation. J. Bacteriol..

[74] Sendecki A.M., Poyton M.F., Baxter A.J., Yang T., Cremer P.S. (2017). Supported lipid bilayers with phosphatidylethanolamine as the major component. Langmuir.

[75] Stenbæk J., Löf D., Falkman P., Jensen B., Cárdenas M. (2017). An alternative anionic bio-sustainable anti-fungal agent: Investigation of its mode of action on the fungal cell membrane. J. Colloid Interface Sci..

[76] Wang K.F., Nagarajan R., Camesano T.A. (2014). Antimicrobial peptide alamethicin insertion into lipid bilayer: A QCM-D exploration. Colloids Surf. B Biointerfaces.

[77] Lind T.K., Caárdenas M., Wacklin H.P. (2014). Formation of supported lipid bilayers by vesicle fusion: Effect of deposition temperature. Langmuir.

[78] Girard-Egrot A.P., Blum L.J. (2007). Langmuir-Blodgett technique for synthesis of biomimetic lipid membranes. Nanobiotechnology of Biomimetic Membranes.

[79] Plant A.L. (1999). Supported hybrid bilayer membranes as rugged cell membrane mimics. Langmuir.

[80] Hillebrandt H., Tanaka M., Sackmann E. (2002). A novel membrane charge sensor: Sensitive detection of surface charge at polymer/lipid composite films on indium tin oxide electrodes. J. Phys. Chem. B.

[81] Kiessling V., Tamm L.K. (2003). Measuring distances in supported bilayers by fluorescence interference-contrast microscopy: Polymer supports and SNARE proteins. Biophys. J..

[82] Groves J.T., Ulman N., Boxer S.G. (1997). Micropatterning fluid lipid bilayers on solid supports. Science.

[83] Andersson J., Fuller M.A., Wood K., Holt S.A., Köper I. (2018). A tethered bilayer lipid membrane that mimics microbial membranes. Phys. Chem. Chem. Phys..

[84] Kulkarni H.M., Nagaraj R., Jagannadham M.V. (2015). Protective role of E. coli outer membrane vesicles against antibiotics. Microbiol. Res..

[85] Arya S.S., Sharma M.M., Rookes J.E., Cahill D.M., Lenka S.K. (2021). Vanilla modulates the activity of antibiotics and inhibits efflux pumps in drug-resistant Pseudomonas aeruginosa. Biologia.

[86] Pitsalidis C., Pappa A.-M., Boys A.J., Fu Y., Moysidou C.-M., van Niekerk D., Saez J., Savva A., Iandolo D., Owens R.M. (2021). Organic bioelectronics for in vitro systems. Chem. Rev..

[87] Lu Z., van Niekerk D., Savva A., Kallitsis K., Thiburce Q., Salleo A., Pappa A.-M., Owens R.M. (2022). Understanding electrochemical properties of supported lipid bilayers interfaced with organic electronic devices. J. Mater. Chem. C.

[88] Jayaram A., Pappa A.-M., Ghosh S., Manzer Z., Traberg W., Knowles T., Daniel S., Owens R. (2021). Biomembranes in bioelectronic sensing. Trends Biotechnol..

[89] Pappa A.-M., Liu H.-Y., Traberg-Christensen W., Thiburce Q., Savva A., Pavia A., Salleo A., Daniel S., Owens R.M. (2020). Optical and electronic ion channel monitoring from native human membranes. ACS Nano.

[90] Su H., Liu H.-Y., Pappa A.-M., Hidalgo T.C., Cavassin P., Inal S., Owens R.M., Daniel S. (2019). Facile generation of biomimetic-supported lipid bilayers on conducting polymer surfaces for membrane biosensing. ACS Appl. Mater. Interfaces.

[91] Rivnay J., Inal S., Salleo A., Owens R.M., Berggren M., Malliaras G.G. (2018). Organic electrochemical transistors. Nat. Rev. Mater..

[92] Pitsalidis C., Pappa A.M., Porel M., Artim C.M., Faria G.C., Duong D.D., Alabi C.A., Daniel S., Salleo A., Owens R.M. (2018). Biomimetic electronic devices for measuring bacterial membrane disruption. Adv. Mater..

[93] Lubrano C., Matrone G.M., Iaconis G., Santoro F. (2020). New frontiers for selective biosensing with biomembrane-based organic transistors. ACS Nano.

[94] Ghosh S., Mohamed Z., Shin J.-H., Naser S.F.B.E., Bali K., Dörr T., Owens R.M., Salleo A., Daniel S. (2022). Impedance sensing of antibiotic interactions with a pathogenic E. coli outer membrane supported bilayer. Biosens. Bioelectron..

[95] Mohamed Z., Shin J.-H., Ghosh S., Sharma A.K., Pinnock F., Bint E Naser Farnush S., Dorr T., Daniel S. (2021). Clinically relevant bacterial outer membrane models for antibiotic screening applications. ACS Infect. Dis..

[96] Epand R.M., Walker C., Epand R.F., Magarvey N.A. (2016). Molecular mechanisms of membrane targeting antibiotics. Biochim. Biophys. Acta (BBA)-Biomembr..

[97] Ciumac D., Gong H., Hu X., Lu J.R. (2019). Membrane targeting cationic antimicrobial peptides. J. Colloid Interface Sci..

[98] Rashki S., Asgarpour K., Tarrahimofrad H., Hashemipour M., Ebrahimi M.S., Fathizadeh H., Khorshidi A., Khan H., Marzhoseyni Z., Salavati-Niasari M. (2021). Chitosan-based nanoparticles against bacterial infections. Carbohydr. Polym..

[99] Mela I., Vallejo-Ramirez P.P., Makarchuk S., Christie G., Bailey D., Henderson R.M., Sugiyama H., Endo M., Kaminski C.F. (2020). DNA nanostructures for targeted antimicrobial delivery. Angew. Chem..

[100] Bali K., Mohamed Z., Scheeder A., Pappa A.-M., Daniel S., Kaminski C.F., Owens R.M., Mela I. (2022). Nanoscale Features of Tunable Bacterial Outer Membrane Models Revealed by Correlative Microscopy. Langmuir.

[101] Chen Q., Rozovsky S., Chen W. (2017). Engineering multi-functional bacterial outer membrane vesicles as modular nanodevices for biosensing and bioimaging. Chem. Commun..

[102] Novikova O., Naberezhnykh G., Sergeev A. (2021). Nanostructured Biosensors Based on Components of Bacterial Membranes. Biophysics.

[103] Ryu H., Fuwad A., Yoon S., Jang H., Lee J.C., Kim S.M., Jeon T.-J. (2019). Biomimetic membranes with transmembrane proteins: State-of-the-art in transmembrane protein applications. Int. J. Mol. Sci..

[104] Kim I., Kwon D., Lee D., Lee G., Yoon D.S. (2019). Permselective glucose sensing with GLUT1-rich cancer cell membranes. Biosens. Bioelectron..

[105] Kim I., Kwon D., Lee D., Lee T.H., Lee J.H., Lee G., Yoon D.S. (2018). A highly permselective electrochemical glucose sensor using red blood cell membrane. Biosens. Bioelectron..

[106] Suri M., Mohamed Z., Bint E Naser S.F., Mao X., Chen P., Daniel S., Hanrath T. (2022). Bioelectronic Platform to Investigate Charge Transfer between Photoexcited Quantum Dots and Microbial Outer Membranes. ACS Appl. Mater. Interfaces.

[107] Kaparakis-Liaskos M., Ferrero R.L. (2015). Immune modulation by bacterial outer membrane vesicles. Nat. Rev. Immunol..

[108] Qasim M., Wrage M., Nüse B., Mattner J. (2022). Shigella Outer Membrane Vesicles as Promising Targets for Vaccination. Int. J. Mol. Sci..

[109] Bjune G., Høiby E., Grønnesby J., Arnesen Ø., Fredriksen J.H., Lindbak A., Nøkleby H., Rosenqvist E., Solberg L., Closs O. (1991). Effect of outer membrane vesicle vaccine against group B meningococcal disease in Norway. Lancet.

[110] van der Ley P., van den Dobbelsteen G. (2011). Next-generation outer membrane vesicle vaccines against Neisseria meningitidis based on nontoxic LPS mutants. Hum. Vaccines.

[111] Gerritzen M.J., Stangowez L., van de Waterbeemd B., Martens D.E., Wijffels R.H., Stork M. (2019). Continuous production of Neisseria meningitidis outer membrane vesicles. Appl. Microbiol. Biotechnol..

[112] Durand V., MacKenzie J., De Leon J., Mesa C., Quesniaux V., Montoya M., Le Bon A., Wong S.Y. (2009). Role of lipopolysaccharide in the induction of type I interferon-dependent cross-priming and IL-10 production in mice by meningococcal outer membrane vesicles. Vaccine.

[113] Quakyi E.K., Frasch C.E., Buller N., Tsai C.-M. (1999). Immunization with meningococcal outer-membrane protein vesicles containing lipooligosaccharide protects mice against lethal experimental group B Neisseria meningitidis infection and septic shock. J. Infect. Dis..

[114] Nagaputra J.C., Rollier C.S., Sadarangani M., Hoe J.C., Mehta O.H., Norheim G., Saleem M., Chan H., Derrick J.P., Feavers I. (2014). Neisseria meningitidis native outer membrane vesicles containing different lipopolysaccharide glycoforms as adjuvants for meningococcal and nonmeningococcal antigens. Clin. Vaccine Immunol..

[115] Long Q., Zheng P., Zheng X., Li W., Hua L., Yang Z., Huang W., Ma Y. (2022). Engineered bacterial membrane vesicles are promising carriers for vaccine design and tumor immunotherapy. Adv. Drug Deliv. Rev..

[116] Olaya-Abril A., Prados-Rosales R., McConnell M.J., Martín-Peña R., González-Reyes J.A., Jiménez-Munguía I., Gómez-Gascón L., Fernández J., Luque-García J.L., García-Lidón C. (2014). Characterization of protective extracellular membrane-derived vesicles produced by Streptococcus pneumoniae. J. Proteom..

[117] Wang X., Thompson C.D., Weidenmaier C., Lee J.C. (2018). Release of Staphylococcus aureus extracellular vesicles and their application as a vaccine platform. Nat. Commun..

[118] Li W., Hu Y., Zhang Q., Hua L., Yang Z., Ren Z., Zheng X., Huang W., Ma Y. (2021). Development of drug-resistant Klebsiella pneumoniae vaccine via novel vesicle production technology. ACS Appl. Mater. Interfaces.

[119] Sinha R., Howlader D.R., Ta A., Mitra S., Das S., Koley H. (2017). Retinoic acid pre-treatment down regulates V. cholerae outer membrane vesicles induced acute inflammation and enhances mucosal immunity. Vaccine.

[120] Ito S., Nakamura J., Fukuta M., Ura T., Teshigawara T., Fukushima J., Mizuki N., Okuda K., Shimada M. (2021). Prophylactic and therapeutic vaccine against Pseudomonas aeruginosa keratitis using bacterial membrane vesicles. Vaccine.

[121] Huang W., Zhang Q., Li W., Chen Y., Shu C., Li Q., Zhou J., Ye C., Bai H., Sun W. (2019). Anti-outer membrane vesicle antibodies increase antibiotic sensitivity of pan-drug-resistant Acinetobacter baumannii. Front. Microbiol..

[122] Irene C., Fantappiè L., Caproni E., Zerbini F., Anesi A., Tomasi M., Zanella I., Stupia S., Prete S., Valensin S. (2019). Bacterial outer membrane vesicles engineered with lipidated antigens as a platform for Staphylococcus aureus vaccine. Proc. Natl. Acad. Sci. USA.

[123] Gerritzen M.J., Martens D.E., Wijffels R.H., van der Pol L., Stork M. (2017). Bioengineering bacterial outer membrane vesicles as vaccine platform. Biotechnol. Adv..

[124] Wai S.N., Lindmark B., Söderblom T., Takade A., Westermark M., Oscarsson J., Jass J., Richter-Dahlfors A., Mizunoe Y., Uhlin B.E. (2003). Vesicle-mediated export and assembly of pore-forming oligomers of the enterobacterial ClyA cytotoxin. Cell.

[125] Wo J., Lv Z.-Y., Sun J.-N., Tang H., Qi N., Ye B.-C. (2022). Engineering Probiotic-derived Outer Membrane Vesicles as Functional Vaccine Carriers to Enhance Immunity against SARS-CoV-2. Iscience.

[126] Gaspar E.B., Prudencio C.R., De Gaspari E. (2021). Experimental studies using OMV in a new platform of SARS-CoV-2 vaccines. Hum. Vaccines Immunother..

[127] Thapa H.B., Müller A.M., Camilli A., Schild S. (2021). An intranasal vaccine based on outer membrane vesicles against SARS-CoV-2. Front. Microbiol..

[128] Gilmore W.J., Johnston E.L., Zavan L., Bitto N.J., Kaparakis-Liaskos M. (2021). Immunomodulatory roles and novel applications of bacterial membrane vesicles. Mol. Immunol..

[129] Çelik P.A., Derkuş B., Erdoğan K., Barut D., Manga E.B., Yıldırım Y., Pecha S., Çabuk A. (2022). Bacterial membrane vesicle functions, laboratory methods, and applications. Biotechnol. Adv..

[130] Wu G., Ji H., Guo X., Li Y., Ren T., Dong H., Liu J., Liu Y., Shi X., He B. (2020). Nanoparticle reinforced bacterial outer-membrane vesicles effectively prevent fatal infection of carbapenem-resistant Klebsiella pneumoniae. Nanomed. Nanotechnol. Biol. Med..

[131] Arya S.S., Rookes J.E., Cahill D.M., Lenka S.K. (2022). Chitosan nanoparticles and their combination with methyl jasmonate for the elicitation of phenolics and flavonoids in plant cell suspension cultures. Int. J. Biol. Macromol..

[132] Arya S.S., Rookes J.E., Cahill D.M., Lenka S.K. (2021). Vanillin: A review on the therapeutic prospects of a popular flavouring molecule. Adv. Tradit. Med..

[133] Zhang X., Xu X., Li Y., Hu C., Zhang Z., Gu Z. (2018). Virion-like membrane-breaking nanoparticles with tumor-activated cell-and-tissue dual-penetration conquer impermeable cancer. Adv. Mater..

[134] Subramaniam S., Joyce P., Thomas N., Prestidge C.A. (2021). Bioinspired drug delivery strategies for repurposing conventional antibiotics against intracellular infections. Adv. Drug Deliv. Rev..

[135] Gao W., Fang R.H., Thamphiwatana S., Luk B.T., Li J., Angsantikul P., Zhang Q., Hu C.-M.J., Zhang L. (2015). Modulating antibacterial immunity via bacterial membrane-coated nanoparticles. Nano Lett..

[136] Zhang Y., Chen Y., Lo C., Zhuang J., Angsantikul P., Zhang Q., Wei X., Zhou Z., Obonyo M., Fang R.H. (2019). Inhibition of pathogen adhesion by bacterial outer membrane-coated nanoparticles. Angew. Chem. Int. Ed..

[137] Adriani R., Gargari S.L.M., Nazarian S., Sarvary S., Noroozi N. (2018). Immunogenicity of Vibrio cholerae outer membrane vesicles secreted at various environmental conditions. Vaccine.

[138] Gao F., Xu L., Yang B., Fan F., Yang L. (2018). Kill the real with the fake: Eliminate intracellular Staphylococcus aureus using nanoparticle coated with its extracellular vesicle membrane as active-targeting drug carrier. ACS Infect. Dis..

[139] Wu Y., Deng G., Song Z., Zhang K., Deng J., Jiang K., Han H. (2022). Enhancing antibacterial immunotherapy for bacterial pneumonia via nanovaccines coated with outer membrane vesicles. Chem. Eng. J..

[140] Chen G., Bai Y., Li Z., Wang F., Fan X., Zhou X. (2020). Bacterial extracellular vesicle-coated multi-antigenic nanovaccines protect against drug-resistant Staphylococcus aureus infection by modulating antigen processing and presentation pathways. Theranostics.

[141] Wu S., Huang Y., Yan J., Li Y., Wang J., Yang Y.Y., Yuan P., Ding X. (2021). Bacterial outer membrane-coated mesoporous silica nanoparticles for targeted delivery of antibiotic rifampicin against Gram-negative bacterial infection in vivo. Adv. Funct. Mater..

[142] Huang W., Zhang Q., Li W., Yuan M., Zhou J., Hua L., Chen Y., Ye C., Ma Y. (2020). Development of novel nanoantibiotics using an outer membrane vesicle-based drug efflux mechanism. J. Control. Release.

[143] Qin J., Yang T., Li J., Zhan G., Li X., Wei Z., Chen Z., Zheng W., Chen H., Yang X. (2022). Bacterial outer membrane vesicle-templated biomimetic nanoparticles for synergistic photothermo-immunotherapy. Nano Today.

[144] Patel R.B., Ye M., Carlson P.M., Jaquish A., Zangl L., Ma B., Wang Y., Arthur I., Xie R., Brown R.J. (2019). Development of an in situ cancer vaccine via combinational radiation and bacterial-membrane-coated nanoparticles. Adv. Mater..

[145] Gao C., Wang Q., Li J., Kwong C.H., Wei J., Xie B., Lu S., Lee S.M., Wang R. (2022). In vivo hitchhiking of immune cells by intracellular self-assembly of bacteria-mimetic nanomedicine for targeted therapy of melanoma. Sci. Adv..

[146] Arya S.S., Lenka S.K., Cahill D.M., Rookes J.E. (2021). Designer nanoparticles for plant cell culture systems: Mechanisms of elicitation and harnessing of specialized metabolites. BioEssays.

[147] Shukla R., Bansal V., Chaudhary M., Basu A., Bhonde R.R., Sastry M. (2005). Biocompatibility of gold nanoparticles and their endocytotic fate inside the cellular compartment: A microscopic overview. Langmuir.

[148] Arya S.S., Rookes J.E., Cahill D.M., Lenka S.K. (2022). Reduced Genotoxicity of Gold Nanoparticles with Protein Corona in Allium cepa. Front. Bioeng. Biotechnol..

[149] Arya S., Sonawane H., Math S., Tambade P., Chaskar M., Shinde D. (2021). Biogenic titanium nanoparticles (TiO_2_NPs) from Tricoderma citrinoviride extract: Synthesis, characterization and antibacterial activity against extremely drug-resistant Pseudomonas aeruginosa. Int. Nano Lett..

[150] Fu Q., Lv P., Chen Z., Ni D., Zhang L., Yue H., Yue Z., Wei W., Ma G. (2015). ProGrammed co-delivery of paclitaxel and doxorubicin boosted by camouflaging with erythrocyte membrane. Nanoscale.

[151] Fang R.H., Kroll A.V., Gao W., Zhang L. (2018). Cell membrane coating nanotechnology. Adv. Mater..

[152] Kroll A.V., Fang R.H., Zhang L. (2017). Biointerfacing and applications of cell membrane-coated nanoparticles. Bioconjug. Chem..

[153] Zou D., Wu Z., Yi X., Hui Y., Yang G., Liu Y., Tengjisi, Wang H., Brooks A., Wang H. (2023). Nanoparticle elasticity regulates the formation of cell membrane-coated nanoparticles and their nano-bio interactions. Proc. Natl. Acad. Sci. USA.

[154] Fang R.H., Gao W., Zhang L. (2022). Targeting drugs to tumours using cell membrane-coated nanoparticles. Nat. Rev. Clin. Oncol..

[155] Zou M.-Z., Li Z.-H., Bai X.-F., Liu C.-J., Zhang X.-Z. (2021). Hybrid vesicles based on autologous tumor cell membrane and bacterial outer membrane to enhance innate immune response and personalized tumor immunotherapy. Nano Lett..

[156] Fang L., Zhao Z., Wang J., Zhang P., Ding Y., Jiang Y., Wang D., Li Y. (2020). Engineering autologous tumor cell vaccine to locally mobilize antitumor immunity in tumor surgical bed. Sci. Adv..

[157] Chen D.J., Osterrieder N., Metzger S.M., Buckles E., Doody A.M., DeLisa M.P., Putnam D. (2010). Delivery of foreign antigens by engineered outer membrane vesicle vaccines. Proc. Natl. Acad. Sci. USA.

[158] Kim O.Y., Park H.T., Dinh N.T.H., Choi S.J., Lee J., Kim J.H., Lee S.-W., Gho Y.S. (2017). Bacterial outer membrane vesicles suppress tumor by interferon-γ-mediated antitumor response. Nat. Commun..

[159] Wang D., Liu C., You S., Zhang K., Li M., Cao Y., Wang C., Dong H., Zhang X. (2020). Bacterial vesicle-cancer cell hybrid membrane-coated nanoparticles for tumor specific immune activation and photothermal therapy. ACS Appl. Mater. Interfaces.

[160] Chen L., Qin H., Zhao R., Zhao X., Lin L., Chen Y., Lin Y., Li Y., Qin Y., Li Y. (2021). Bacterial cytoplasmic membranes synergistically enhance the antitumor activity of autologous cancer vaccines. Sci. Transl. Med..

[161] Park J.H., Mohapatra A., Zhou J., Holay M., Krishnan N., Gao W., Fang R.H., Zhang L. (2022). Virus-mimicking cell membrane-coated nanoparticles for cytosolic delivery of mRNA. Angew. Chem..

[162] Huang Y., Beringhs A.O.R., Chen Q., Song D., Chen W., Lu X., Fan T.-H., Nieh M.-P., Lei Y. (2019). Genetically engineered bacterial outer membrane vesicles with expressed nanoluciferase reporter for in vivo bioluminescence kinetic modeling through noninvasive imaging. ACS Appl. Bio. Mater..

[163] Li Z., Zhang Y., Zhu C., Guo T., Xia Q., Hou X., Liu W., Feng N. (2020). Folic acid modified lipid-bilayer coated mesoporous silica nanoparticles co-loading paclitaxel and tanshinone IIA for the treatment of acute promyelocytic leukemia. Int. J. Pharm..

[164] Pichler H., Emmerstorfer-Augustin A. (2018). Modification of membrane lipid compositions in single-celled organisms–From basics to applications. Methods.

[165] Price N.L., Goyette-Desjardins G., Nothaft H., Valguarnera E., Szymanski C.M., Segura M., Feldman M.F. (2016). Glycoengineered outer membrane vesicles: A novel platform for bacterial vaccines. Sci. Rep..

[166] Valguarnera E., Feldman M.F. (2017). Glycoengineered outer membrane vesicles as a platform for vaccine development. Methods in Enzymology.

[167] Manzer Z.A., Ghosh S., Jacobs M.L., Krishnan S., Zipfel W.R., Piñeros M., Kamat N.P., Daniel S. (2021). Cell-Free Synthesis of a Transmembrane Mechanosensitive Channel Protein into a Hybrid-Supported Lipid Bilayer. ACS Appl. Bio. Mater..

[168] Kim D.-K., Lee J., Kim S.R., Choi D.-S., Yoon Y.J., Kim J.H., Go G., Nhung D., Hong K., Jang S.C. (2015). EVpedia: A community web portal for extracellular vesicles research. Bioinformatics.

